# Some approximation results for mild solutions of stochastic fractional order evolution equations driven by Gaussian noise

**DOI:** 10.1007/s40072-022-00250-0

**Published:** 2022-04-26

**Authors:** K. Fahim, E. Hausenblas, M. Kovács

**Affiliations:** 1 Department of Mathematics, Institut Teknologi Sepuluh Nopember, Kampus ITS Sukolilo, Surabaya, 60111 Indonesia; 2 Department of Mathematics, Montanuniversity Leoben, 8700 Leoben, Austria; 3 Faculty of Information Technology and Bionics, Pázmány Péter Catholic University, Budapest, Hungary; 4 Department of Differential Equations, Faculty of Natural Sciences, Budapest University of Technology and Economics, Müegyetem rkp. 3., Budapest 1111, Hungary; 5 Chalmers University of Technology, Gothenburg, Sweden

**Keywords:** Stochastic partial differential equation, Stochastic integro-differential equation, Wiener process, Fractal Wiener process, Stochastic Volterra equation, Finite element method, Spectral Galerkin method, Fractional partial differential equation, 45D05, 60H15, 60H20, 60G22, 65L03

## Abstract

We investigate the quality of space approximation of a class of stochastic integral equations of convolution type with Gaussian noise. Such equations arise, for example, when considering mild solutions of stochastic fractional order partial differential equations but also when considering mild solutions of classical stochastic partial differential equations. The key requirement for the equations is a smoothing property of the deterministic evolution operator which is typical in parabolic type problems. We show that if one has access to nonsmooth data estimates for the deterministic error operator together with its derivative of a space discretization procedure, then one obtains error estimates in pathwise Hölder norms with rates that can be read off the deterministic error rates. We illustrate the main result by considering a class of stochastic fractional order partial differential equations and space approximations performed by spectral Galerkin methods and finite elements. We also improve an existing result on the stochastic heat equation.

## Introduction

Let *H* be a real separable Hilbert space and let $W_H$ be a *H*-cylindrical Wiener process on a complete, filtered probability space $(\Omega ,\mathcal {F},(\mathcal {F}_t)_{t\ge 0},{\mathbb {P}})$ with respect to the filtration $(\mathcal {F}_t)_{t\ge 0}$. To be more precise, we assume that $(\mathcal {F}_t)_{t\ge 0}$ satisfies the usual conditions, which are, $(\mathcal {F}_t)_{t\ge 0}$ is right-continuous and $\mathcal {F}_0$ contains all ${\mathbb {P}}$-nullsets of $\mathcal {F}$. Let $H_i$, $i=0,1,2$ be real separable Hilbert spaces to be specified later on but they are typically associated with fractional powers of a linear operator. We consider integral equations of the form

1 $$\begin{aligned} U(t)=X^0(t)+\int _{0}^{t}S^2(t-s)F(s,U(s))\,ds+\int _{0}^{t}S^1(t-s)G(s,U(s))\,dW_H(s).\nonumber \\ \end{aligned}$$

Here, the non-linear functions $G:[0,T]\times H_0\rightarrow L_{HS}(H,H_1)$, where $L_{HS}(H,H_1)$ denotes the space of Hilbert-Schmidt operators form *H* to $H_1$, and $F:[0,T]\times H_0\rightarrow H_2$ are assumed to satisfy global Lipschitz and linear growth conditions. The operator families $S^i(t):H_i\rightarrow H_0$, $i=1,2,$ are assumed to admit certain smoothing estimates for $t>0$.

A typical example where the integral Eq. (1) arises is when defining mild solutions of fractional order equations of the form [23],

2 $$\begin{aligned} \begin{aligned} U(t)&= U_0+tU_1-A\int _0 ^t \frac{(t-s)^{\alpha -1}}{\Gamma (\alpha )} U(s)\,ds \\&{}\quad + \int _0 ^t \frac{(t-s)^{\kappa -1}}{\Gamma (\kappa )} F(s,U(s))\,ds \\&{}\quad + \int _0^ t \frac{(t-s)^{\beta -1}}{\Gamma (\beta )} \, G(s,U(s))\,dW_H(s);\quad t\in [0,T], \end{aligned} \end{aligned}$$

see Sect. 3.1 for more details. Here, *A* is a densely defined, possibly unbounded, non-negative operator on the Hilbert space $H_0$, $\alpha \in (0,2)$, $\beta >\frac{1}{2}$ and $\kappa >0$. The restriction $\beta >\frac{1}{2}$ is needed otherwise the stochastic integral does not make sense even for constant *G* as for $\beta \le \frac{1}{2}$ the function $t\rightarrow t^{\beta -1}$ is not square integrable on [0, *T*]. For $\alpha \in (0,1)$ (and $U_1=0$ in this case), Eq. (2) becomes a fractional stochastic heat equation, for $\alpha \in (1,2)$ Eq. (2) becomes a fractional stochastic wave equation.

Time fractional stochastic heat type equations might be used to model phenomena with random effects with thermal memory [48]. In its simplest form, the fractional stochastic heat equation has the form

3 $$\begin{aligned} dU+AD_t^{1-\alpha }(U)dt=F(U)dt+G(U)dW_H(t); \,U(0)=U_0,\,\alpha \in (0,1), \end{aligned}$$

where

$$\begin{aligned} D_t^{1-\alpha }(U)(t)=\frac{1}{\Gamma (\alpha )}\frac{d}{dt}\int _0^t(t-s)^{\alpha -1}U(s)\,d s,\,\alpha \in (0,1). \end{aligned}$$

Eq. (3) corresponds to (2) with $\beta =\kappa =1$, $\alpha \in (0,1)$ and $U_1=0$.

Time fractional stochastic wave type equations may be used to model random forcing effects in viscoelastic materials which exhibit a simple power-law creep behaviour [13, 38]. The simplest form of the fractional stochastic wave equation takes the form

4 $$\begin{aligned} dU+AI^{\alpha -1}(U)dt=U_1dt+F(U)dt+G(U)dW_H(t); \,U(0)=U_0,\,\alpha \in (1,2),\nonumber \\ \end{aligned}$$

where

$$\begin{aligned} I^{\alpha -1}(U)(t)=\frac{1}{\Gamma (\alpha -1)}\int _0^t(t-s)^{\alpha -2}U(s)\,ds,\, \alpha >1, \end{aligned}$$

and $U_1$ is the initial data for ${\dot{U}}$. Equation (4) corresponds to (2) with $\beta =\kappa =1$ and $\alpha \in (1,2)$.

In both cases the parameters $\beta$ and $\kappa$ can be used to model the time-regularity of the stochastic, respectively, the deterministic feedback. For example, when $\beta <1$, then the driving process is rougher than the Wiener process, while if $\beta >1$, it is smoother. It is important to note that while the parameter choice in (2) corresponding to $\alpha =\beta =\kappa =1$ is the standard stochastic heat equation, the parameter choice $\beta =\kappa =1$ and $\alpha = 2$ does not result in the standard stochastic wave equation but in something much more irregular as in this case the noise will drive ${\dot{U}}$ and not ${\ddot{U}}$. The standard stochastic wave equation would correspond to the choice $\beta =\kappa =2$ and $\alpha = 2$. This case is not covered by our paper, since in our setting crucial estimates has constants blowing up as $\alpha \rightarrow 2$. In particular, the constants in the fundamental regularity estimates (20) and (21) will blow up, and therefore we cannot say anything for the limiting case $\alpha =2$ by taking $\alpha \rightarrow 2$.

Our aim is to approximate stochastic integro-differential equations of the type (1) and derive error estimates in pathwise Hölder norms in time. To this end we consider approximations of (1) given by the following integral equation

$$\begin{aligned} U_n(t)&=X_n^0(t)+\int _{0}^{t}S_n^2(t-s)F(s,U_n(s))\,ds+\int _{0}^{t}S_n^1(t-s)G(s,U_n(s))\,dW_H(s). \end{aligned}$$

Here, the approximation $U_n$ can typically be a spatial approximation derived via a spectral Galerkin or a continuous finite element method. The main purpose of our work is to derive rates of convergence of the strong error over $C^\gamma ([0,T];H_0)$; that is, we derive error estimates for $U-U_n$ in $L^p(\Omega ;C^\gamma ([0,T];H_0))$. Here, for a function $f:[0,T]\rightarrow E$, where *E* is a Banach space, the Hölder seminorm is defined by

$$\begin{aligned} \Vert f\Vert _{C^\gamma ([0,T];E)}=\sup _{{\mathop {t\ne s}\limits ^{t,s \in [0,T]}}}\frac{\Vert f(t)-f(s)\Vert _E}{|t-s|^\gamma },\,\gamma \in (0,1). \end{aligned}$$

In particular, we derive a rate of convergence of the strong error over $C([0,T];H_0)$ (the space of $H_0$-valued continuous functions on [0, *T*] equipped with the supremum norm); that is, an error estimate for $U-U_n$ in $L^p(\Omega ;C([0,T];H_0))$. These error estimates are derived given that one has access to deterministic nonsmooth error estimates for $S^i-S^i_n$ and $\frac{d}{dt}(S^i-S^i_n)$. The main point is that the rate of convergence in these norms can be directly read off the deterministic error rates. While traditionally error estimates for $\frac{d}{dt}(S^i-S^i_n)$ are seldom considered they are not out of reach in many cases (see, for example, [55, Theorem 3.4], for finite elements for parabolic problems). We demonstrate by two examples how to obtain such estimates for fractional order equations both for spectral Galerkin and for a standard continuous finite element method. In general, when $S^i$ are resolvent families for certain parabolic integro-differential problems arising, for example, in viscoelasticity, [3, 13, 38, 52], these nonsmooth data estimates are direct consequences of the smoothing property of the resolvent family of the linear deterministic problem, at least for the spectral Galerkin method.

Our motivation for considering estimates in such norms is twofold. Firstly, estimates with respect to the $L^p(\Omega ;C([0,T];H_0))$-norm are useful for using standard localization arguments [29, 51] in order to extend approximation results for equations with globally Lipschitz continuous nonlinearities to results for equations with nonlinearities that are only Lipschitz continuous on bounded sets. We refer to [18, Section 4] for further details in the semigroup case. Secondly, as Remark 2.10 shows, the processes *U* and $U_n$ can be viewed as random variables in $L^p(\Omega ;C^\gamma ([0,T];H_0))$ and therefore it natural to consider the approximation error in the corresponding norm. For further applications of approximations in Hölder norms we refer to [18] and [4].

Finally, we would like to emphasize that the derivation of error estimates in the $L^p(\Omega ;C([0,T];H_0))$-norm is usually a nontrivial task even when the operator family $S^1$ is a semigroup. This is because, in general, the stochastic convolution appearing in (1) fails to be a semimartingale and hence Doob’s maximal inequality cannot be applied to obtain estimates with respect to the $L^p(\Omega ;C([0,T];H_0))$-norm. In case the operator family $S^1$ is a semigroup one may employ the factorization method of Da Prato, Kwapien and Zabczyk [12] directly to obtain such estimates, for an instance, see, for example, [37]. However, when the semigroup property does not hold; that is, there is a nontrivial memory effect in the equation, then even this approach fails.

### The state of the art

For analytical results, such as existence, uniqueness and regularity of various stochastic Volterra-type integro-differential equations driven by Wiener noise we refer to [3, 5, 8–10, 13, 14, 32–34] and for results concerning asymptotic behaviour of solutions to [7, 24]. The particular case of fractional order equations driven by Wiener noise are considered in [11, 21–23, 35, 43, 48]. Various integro-differential equations driven by Lévy noise are analysed in [20, 30] with the particular case of fractional order equations in [6]. Finally a class of linear Volterra integro-differential equations driven by fractional Brownian motion are investigated in [53, 54].

The main purpose of our work is to derive rates of convergence for space approximations. Here, we consider the strong error over $C^\gamma ([0,T];H_0)$ and $C([0,T];H_0)$, that is, we derive error estimates in $L^p(\Omega ;C^\gamma ([0,T];H_0))$ and $L^p(\Omega ;C([0,T];H_0))$. To our knowledge all existing work on the numerical analysis of stochastic fractional order differential equations are considering a much weaker error measure; that is, the error measure $\sup _{t\in [0,T]}{\mathbb E}\Vert U(t)-U_n(t)\Vert _{H_0}^p$ (mainly for $p=2$), see, for example, [26, 27, 31, 38, 40, 56], or the weak error [1, 31, 39, 41]. For similar works in the setting of abstract evolution equations without memory kernel we refer to [16, 17], and [18].

### The structure of the paper

The paper is organized as follows. In Sect. 2 we introduce the abstract setting and the assumptions which we will use. Here we illustrate the applicability of our setting by several examples. In Lemma 2.9 we state and prove a basic existence and uniqueness result for the solution of (1) and specify the time-regularity of the solution in Remark 2.10. Our main abstract approximation result estimating the difference $U-U_n$ in $L^p(\Omega ;C^\gamma ([0,T];H_0))$ is contained in Theorem 2.11 while in $L^p(\Omega ;C([0,T];H_0))$ in Corollary 2.12. In Sect. 3 we apply Theorem 2.11 and Corollary 2.12 to two typical space discretization schemes. In particular, in Subsection 3.1 we consider the general fractional-order Eq. (2) (with time-independent coefficients, for simplicity) and its spectral Galerkin approximation and apply Theorem 2.11 and Corollary 2.12 to obtain rates of convergence depending on the parameters in the equation. In Examples 3.6, 3.7, and 3.8 we state the results in some simplified settings for the stochastic heat equation, the fractional stochastic heat and wave equations, respectively. In Subsection 3.2 we consider a fractional stochastic wave equation and its finite element approximation and apply Theorem 2.11 and Corollary 2.12 again to obtain rates of convergence. Here, in Remark 3.13, we point out in which way the stochastic heat equation fits in our abstract framework and we also show that using the setup of the paper one may remove some unnecessary smoothness assumption on *G* which was present in [16, Proposition 4.2]. Finally, in Sect. 4, we present some numerical experiments for a fractional stochastic wave equation to verify the theoretical rates obtained in Subsections 3.1 and 3.2. In particular, in Subsection 4.1, we present some numerical results for the spectral Galerkin approximation and space-time white noise, while in Subsection 4.2, we describe some experiments for the finite element method and trace-class noise.

### Notation

We denote by $\mathbb {R}_0^+$ the set $\{t\in \mathbb {R}:t\ge 0\}$. For Banach spaces *V* and *W* we denote by $\mathcal {L}(V,W)$ the space of bounded linear operators from *V* into *W* endowed with the norm

$$\begin{aligned} \Vert A\Vert _{\mathcal {L}(V,W)} = \inf \{ C \ge 0 : \Vert Av\Vert _W \le C \Vert v\Vert _V \text{ for } \text{ all } v \in V \}, ~\text {for }A\in \mathcal {L}(V,W). \end{aligned}$$

If $V=W$, we write $\mathcal {L}(V)$ for $\mathcal {L}(V,W)$ and we denote the norm by $\Vert \cdot \Vert _{\mathcal {L}(V)}$. For an operator valued function $S:[a,b]\rightarrow \mathcal {L}(V,W)$ we use the notation

$$\begin{aligned} {\dot{S}}(t)v:=\frac{d}{dt}\big (t\mapsto S(t)v\big )(t),\,v\in V, \end{aligned}$$

whenever $t\mapsto S(t)v$ is differentiable at *t*. Furthermore, for two real separable Hilbert spaces *V* and *W*, we denote by $L_{HS}(V,W)$ the space of Hilbert-Schmidt operators from *V* to *W* equipped with the norm

$$\begin{aligned} \Vert T\Vert _{{L_{HS}(V,W)}}^2=\sum \limits _{n=1}^{\infty }\Vert Te_n\Vert _W^2,~\text {for }T\in L_{HS}(V,W), \end{aligned}$$

for an orthonormal basis $$(e_n)\subset V$$. Let *H* be a real, separable, infinite-dimensional Hilbert space and let $$A:D(A)\subseteq H \rightarrow H$$, be an unbounded, self-adjoint, and positive definite operator with compact inverse. For $$\xi \in \mathbb {R}$$ one defines the fractional power $$A^\xi $$ of *A* via the standard spectral functional calculus of *A*. For $$\xi \ge 0$$ we equip $$D(A^\xi )$$, where $$D(A^\xi )$$ denotes the domain of $$A^\xi $$, with the norm $$\Vert x\Vert _{D(A^\xi )}:=\Vert A^\xi x\Vert _H$$, $$x\in A^\xi $$. For $$\delta \ge 0$$, let $$H_{-\delta }^A$$, denote the completion of *H* with respect to the norm $$ \Vert x\Vert _{{-\delta }}:=\Vert A^{-\delta }x\Vert _{H}, $$$$x\in H_{-\delta }^A$$. Let *E* be a Banach space. We denote by $$L^p([0,T];E)$$, $$1\le p<\infty $$, the space of all measurable functions $$f:[0,T]\rightarrow E$$ being $$L^p$$ integrable equipped the with the standard norm

$$\begin{aligned} \Vert f\Vert _{L^p([0,T];E)}:=\Big (\int _0^T \Vert {f(t)} \Vert _E^p~dt\Big )^{1/p}. \end{aligned}$$

Moreover if $p=\infty$, then $L^\infty ([0,T];E)$ denotes the space of all measurable functions *f* from [0, *T*] to *E* being essential bounded in *E* on [0, *T*] equipped with the norm

$$\begin{aligned} \Vert f\Vert _{L^{\infty }([0,T];E)}={\mathop {\mathrm{ess\ sup}}\limits _{t\in [0,T]}}\Vert f(t) \Vert _E. \end{aligned}$$

We denote by *C*([0, *T*]; *E*) the space of continuous functions $f:[0,T]\rightarrow E$ endowed with the usual supremum norm. Let $C^\gamma ([0,T];E)$, $0<\gamma <1$, denote the space of functions $f:[0,T]\rightarrow E$ such that the seminorm

$$\begin{aligned} \Vert f\Vert _{C^\gamma ([0,T];E)}:=\sup _{{\mathop {t\ne s}\limits ^{t,s \in [0,T]}}}\frac{\Vert f(t)-f(s)\Vert _E}{|t-s|^\gamma }<\infty . \end{aligned}$$

Let $(\Omega ,\mathcal {F},\mathbb {P})$ be a probability space and let $L^p(\Omega ;E)$ denote the space of random variables $X:(\Omega ,\mathcal {F})\rightarrow (E,\mathcal {B}(E))$; that is, $\mathcal {F}/\mathcal {B}(E)$-measurable mappings $X:\Omega \rightarrow E$, where $\mathcal {B}(E)$ denotes the Borel $\sigma$-algebra of *E*, such that

$$\begin{aligned} \Vert X\Vert _{L^p(\Omega ;E)}^p=\mathbb {E}\big (\Vert X\Vert _{E}^p\big ) =\int \limits _{\Omega }\Vert X(\omega )\Vert _{E}^p\,d \mathbb {P}(\omega )<\infty . \end{aligned}$$

## The abstract approximation result

Let *H* and $H_0$ be two real separable Hilbert spaces. Let $W_H$ be a *H*-cylindrical Wiener process on a complete, filtered probability space $(\Omega ,\mathcal {F},(\mathcal {F}_t)_{t\ge 0},{\mathbb {P}})$ with respect to the filtration $(\mathcal {F}_t)_{t\ge 0}$, the latter satisfying the usual conditions. We consider the following integral equation

5 $$\begin{aligned} U(t)=X^0(t)+\int _{0}^{t}S^2(t-s)F(s,U(s))\,ds+\int _{0}^{t}S^1(t-s)G(s,U(s))\,dW_H(s).\nonumber \\ \end{aligned}$$

To specify the assumptions on the coefficients *F* and *G*, let us fix two real separable Hilbert spaces $H_1$ and $H_2$. Later on, we will see in the examples that these spaces will be interpolation spaces associated with a linear operator.

### Assumption 2.1

We assume that

1. the mapping $G: [0,T]\times H_0\rightarrow L_{HS}(H,H_1)$ is Lipschitz continuous and of linear growth in the second variable uniformly in [0, *T*]; that is, there is a constant $C_G>0$ such that
  $$\begin{aligned}&\Vert G(t,u)-G(s,v)\Vert _{ L_{HS}(H,H_1)}\le C_G(|t-s|+\Vert u-v\Vert _{H_0}),\,u,v\in H_0, \\&\quad s,t\in [0,T]; \end{aligned}$$
  and
  $$\begin{aligned} \Vert G(t,u)\Vert _{ L_{HS}(H,H_1)}\le C_G(1+\Vert u\Vert _{H_0}),\,u\in H_0, \,t\in [0,T]; \end{aligned}$$
2. the mapping $F: [0,T]\times H_0\rightarrow H_2$ is Lipschitz continuous and of linear growth in the second variable uniformly in [0, *T*]; that is there is a constant $C_F>0$ such that
  $$\begin{aligned} \Vert F(t,u)-F(s,v)\Vert _{H_2}\le C_F(|t-s|+\Vert u-v\Vert _{H_0}),\,u,v\in H_0, \,s,t\in [0,T]; \end{aligned}$$
  and
  $$\begin{aligned} \Vert F(t,u)\Vert _{ H_2}\le C_F(1+\Vert u\Vert _{H_0}),\,u\in H_0, \,t\in [0,T]; \end{aligned}$$
3. the $H_0$-valued process $\{X^0(t)\}_{t\in [0,T]}$ is predictable and, for some $p>2$,
  $$\begin{aligned} X^0\in L^p(\Omega ; L^\infty ([0,T];H_0)). \end{aligned}$$

We note that the *t*-dependence of *F* and *G* may be weakened considerably and they may also be stochastic. Concerning the families $S^i$ we suppose the following.

### Assumption 2.2

We assume that the linear operator families $S^i$ are strongly continuously differentiable as operators from $H_i$ to $H_0$ on (0, *T*), $i=1,2$. Furthermore, we assume that

1. there exists a function $s_1\in L^ 1 ([0,T];\mathbb {R}^+_0)$ and a constant $0<\gamma _1<\frac{1}{2}$ such that
  $$\begin{aligned} t^{\gamma _1} \Vert \dot{S}^1(t) x\Vert _{H_0} + t^{\gamma _1-1} \Vert S^1(t) x\Vert _{H_0}&\le s_1(t)\Vert x\Vert _{H_1}, \, \text {for all } x\in H_1,\,t\in (0,T); \end{aligned}$$
2. there exists a function $s_2\in L^ 1 ([0,T];\mathbb {R}^+_0)$ and a constant $0<\gamma _2<1$ such that
  $$\begin{aligned} t^{\gamma _2} \Vert \dot{S}^2(t) x\Vert _{H_0} + t^{\gamma _2-1} \Vert S^2(t) x\Vert _{H_0}&\le s_2(t)\Vert x\Vert _{H_2}, \, \text {for all } x\in H_2,\,t\in (0,T). \end{aligned}$$

We consider an approximation of (5) given by the following integral equation

6 $$\begin{aligned} U_n(t)=X_n^0(t)+\int _{0}^{t}S_n^2(t-s)F(s,U_n(s))\,ds+\int _{0}^{t}S_n^1(t-s)G(s,U_n(s))\,dW_H(s),\nonumber \\ \end{aligned}$$

where $\{X_n^0(t)\}_{t\in [0,T]}$ is $H_0$-predictable and $X^0_n\in L^p(\Omega ; L^\infty ([0,T];H_0))$. Concerning the approximation we make the following assumptions.

### Assumption 2.3

Let $\gamma _1$ and $\gamma _2$ be as in Assumption 2.2. For bounded functions $r_i:\mathbb {N}\rightarrow \mathbb {R}^ +$, $i=1,2$ consider

$$\begin{aligned} \Psi ^{i}_n(t):= \frac{1}{r_i(n)} \left[ S^i(t)- S^i_n(t)\right] ,\,i=1,2. \end{aligned}$$

We assume that the linear operator families $S^i_n$ are strongly continuously differentiable as operators from $H_i$ to $H_0$ on (0, *T*), $i=1,2$. Furthermore, we assume that

1. there exists a function $h_1\in L ^1 ([0,T];\mathbb {R}^+_0)$ such that for all $n\in \mathbb {N}$ we have
  $$\begin{aligned} t^{\gamma _1} \Vert {\dot{\Psi }}^1_n(t) x\Vert _{H_0} + t^{\gamma _1-1} \Vert \Psi ^1_n(t) x\Vert _{H_0}&\le h_1(t)\Vert x\Vert _{H_1}, \, \text {for all } x\in H_1,\,t\in (0,T); \end{aligned}$$
2. there exists a function $h_2\in L ^1 ([0,T];\mathbb {R}^+_0)$ such that for all $n\in \mathbb {N}$ we have
  $$\begin{aligned} t^{\gamma _2} \Vert {\dot{\Psi }}^2_n(t) x\Vert _{H_0} + t^{\gamma _2-1} \Vert \Psi ^2_n(t) x\Vert _{H_0}&\le h_2(t)\Vert x\Vert _{H_2}, \, \text {for all } x\in H_2,\,t\in (0,T). \end{aligned}$$

Note that Assumptions 2.2 and 2.3 imply that for some $C>0$, for all $n\in {\mathbb {N}}$,

7 $$\begin{aligned} t^{\gamma _1} \Vert \dot{S}_n^1(t) x\Vert _{H_0} + t^{\gamma _1-1} \Vert S_n^1(t) x\Vert _{H_0}&\le (s_1(t)+Ch_1(t))\Vert x\Vert _{H_1}, \end{aligned}$$

for all $x\in H_1$, $t\in (0,T)$, and

8 $$\begin{aligned} t^{\gamma _2} \Vert \dot{S}_n^2(t) x\Vert _{H_0} + t^{\gamma _2-1} \Vert S_n^2(t) x\Vert _{H_0}&\le (s_2(t)+Ch_2(t))\Vert x\Vert _{H_2}, \end{aligned}$$

for all $$x\in H_2$$, $$t\in (0,T)$$.

### Example 2.4

(Stochastic heat equation) In order to illustrate a typical situation of our basic assumptions, we first consider the familiar setting of the heat equation. Let $A:D(A)\subset H_0\rightarrow H_0$ be an unbounded, densely defined, self-adjoint, positive definite operator with compact inverse. Let $\lambda _n$ denote the eigenvalues of *A*, arranged in a non-decreasing order, with corresponding orthonormal eigenbasis $(e_n)\subset H_0$. A typical example is when $H_0=L^2(\mathcal {D})$, where $\mathcal {D}\subset {\mathbb R}^d$ is a bounded domain with smooth or convex polygonal boundary, and $A=-\Delta$ with Dirichlet zero boundary conditions. In this case, we have $S^1(t)=S^2(t)=e^{-tA}$. Typically the spaces $H_1$ and $H_2$ are related to the fractional powers of *A* but for simplicity we take $H_1=H_2=H=H_0$. In this case, Assumptions  2.1 become standard global Lipschitz assumptions on the coefficients in the equation. Furthermore, due to the analyticity of the semigroup $S(t):=e^{-tA}$ one has the well-known smoothing properties

$$\begin{aligned} \Vert A^\xi S(t)x\Vert _{H_0}\le Mt^{-\xi }\Vert x\Vert _{H_0},\quad \Vert A^\xi \dot{S}(t)x\Vert _{H_0}\le Mt^{-\xi -1}\Vert x\Vert _{H_0}, \end{aligned}$$

for $t>0$ and $\xi \ge 0$. Then Assumption 2.2-(1) is satisfied for any $0<\gamma _1<\frac{1}{2}$ for $s_1(t)=Mt^{\gamma _1-1}$ and Assumption 2.2-(2) is satisfied for any $0<\gamma _2<1$ for $s_2(t)=Mt^{\gamma _2-1}$. The simplest example of an approximation procedure that we have in mind is the spectral Galerkin method. We define a family of finite-dimensional subspaces $\{H^n:n\in \mathbb {N}\}$ of $H_0$ by

$$\begin{aligned} {H^n}=\text {span}\{e_1,e_2,\dots ,e_n\} \end{aligned}$$

and define the orthogonal projection

9 $$\begin{aligned} \mathcal {P}_n:H_0\rightarrow {H^n},\quad \mathcal {P}_n x=\sum _{k=1}^n ( x,e_k)_{H_0} e_k,\quad x\in H_0, \end{aligned}$$

where $(\cdot \,,\cdot )_{H_0}$ denotes the inner product of $H_0$. It is easy to see that

$$\begin{aligned} \Vert A^{-\nu }(I-\mathcal {P}_n)\Vert _{\mathcal {L}(H_0)}= \Vert (I-\mathcal {P}_n)A^{-\nu }\Vert _{\mathcal {L}(H_0)} =\sup _{k\ge n+1}\lambda _k^{-\nu } =\lambda _{n+1}^{-\nu },\quad \nu \ge 0. \end{aligned}$$

The approximating operators become

$$\begin{aligned} S^1_n(t)=S^2_n(t)=\mathcal {P}_nS(t)=S(t)\mathcal {P}_n:=S_n(t). \end{aligned}$$

Using eigenfunctions and eigenvalues of *A* we can write

$$\begin{aligned} S_n(t)x=\sum _{k=1}^ne^{-\lambda _k t}(x,e_k)_{H_0}e_k. \end{aligned}$$

For $\nu \ge 0$, we set $r_1(n)=r_2(n)=\lambda _{n+1}^{-\nu }$. We then have

$$\begin{aligned} \Psi ^1_n(t)=\Psi ^2_n(t)=\lambda _{n+1}^{\nu }(S(t)-S_n(t))=\lambda _{n+1}^{\nu }(I-\mathcal {P}_n)S(t) \end{aligned}$$

with

$$\begin{aligned} \Vert \Psi ^i_n(t)x\Vert _{H_0}\le C\Vert A^\nu S(t)x\Vert _{H_0}\le C t^{-\nu }\Vert x\Vert _{H_0},\,i=1,2\text { and }n\in {\mathbb {N}}, \end{aligned}$$

and

$$\begin{aligned} \Vert \dot{\Psi }^i_n(t)x\Vert _{H_0}\le C\Vert A^\nu {\dot{S}}(t)x\Vert _{H_0}\le C t^{-\nu -1}\Vert x\Vert _{H_0},\,i=1,2\text { and }n\in {\mathbb {N}}. \end{aligned}$$

Thus, Assumption 2.3 is satisfied with $r_1(n)=\lambda _{n+1}^{-\nu _1}$ with $\nu _1<\gamma _1$ and $h_1(t)=Ct^{\gamma _1-1-\nu _1}$ and $r_2(n)=\lambda _{n+1}^{-\nu _2}$ with $\nu _2<\gamma _2$ and $h_2(t)=Ct^{\gamma _2-1-\nu _2}$.

### Example 2.5

(Fractional stochastic heat Equation) Here we consider the fractional stochastic heat Eq. (3) with mild solution given by (17) with parameters $\alpha \in (0,1)$, $\beta =\kappa =1$, $u_0=U_0$ and $u_1=0$, where the operator family $S^{\alpha ,\beta }$ is defined by (18) via its Laplace transform. We use the setting of the previous example for *A*, *F* and *G*; that is, consider the global Lipschitz case. In this case we have $S^{1}(t)=S^2(t)=S^{\alpha ,1}(t)$ with smoothing properties specified in Lemma 3.1. In particular, we have

$$\begin{aligned} \Vert A^\xi S^i(t)x\Vert _{H_0}\le Mt^{-\alpha \xi }\Vert x\Vert _{H_0},\quad \Vert A^\xi \dot{S}^i(t)x\Vert _{H_0}\le Mt^{-\alpha \xi -1}\Vert x\Vert _{H_0},\,i=1,2, \end{aligned}$$

for $\xi \in [0,1]$ and $t>0$. Then, Assumption 2.2-(1) is satisfied for any $0<\gamma _1<\frac{1}{2}$ for $s_1(t)=Mt^{\gamma _1-1}$ and Assumption 2.2-(2) is satisfied for any $0<\gamma _2<1$ for $s_2(t)=Mt^{\gamma _2-1}$. The approximating operators in this case become

$$\begin{aligned} S^1_n(t)=S^2_n(t)=\mathcal {P}_nS^{\alpha ,1}(t)=S^{\alpha ,1}(t)\mathcal {P}_n:=S^{\alpha ,1}_n(t). \end{aligned}$$

For $\nu \in [0,1]$, we set $r_1(n)=r_2(n)=\lambda _{n+1}^{-\nu }$. We then have

$$\begin{aligned} \Psi ^1_n(t)=\Psi ^2_n(t)=\lambda _{n+1}^{\nu }(S^{\alpha ,1}(t)-S^{\alpha ,1}_n(t))=\lambda _{n+1}^{\nu }(I-\mathcal {P}_n)S^{\alpha ,1}(t) \end{aligned}$$

with

$$\begin{aligned} \Vert \Psi ^i_n(t)x\Vert _{H_0}\le C\Vert A^\nu S^{\alpha ,1}(t)x\Vert _{H_0}\le C t^{-\alpha \nu }\Vert x\Vert _{H_0},\,i=1,2\text { and }n\in {\mathbb {N}}, \end{aligned}$$

and

$$\begin{aligned} \Vert \dot{\Psi }^i_n(t)x\Vert _{H_0}\le C\Vert A^\nu {\dot{S}}^{\alpha ,1}(t)x\Vert _{H_0}\le C t^{-\alpha \nu -1}\Vert x\Vert _{H_0},\,i=1,2\text { and }n\in {\mathbb {N}}. \end{aligned}$$

Thus, Assumption 2.3 is satisfied with $r_1(n)=\lambda _{n+1}^{-\nu _1}$ with $\nu _1<\frac{\gamma _1}{\alpha }$ and $h_1(t)=Ct^{\gamma _1-1-\alpha \nu _1}$ and $r_2(n)=\lambda _{n+1}^{-\nu _2}$ with $\nu _2<\frac{\gamma _2}{\alpha }$ and $h_2(t)=Ct^{\gamma _2-1-\alpha \nu _2}$. It is important to note that the additional restriction $\max (\nu _1,\nu _2)\le 1$ applies as $S^{\alpha ,1}$ has only the limited smoothing properties shown in Lemma 3.1. This implies that the rates improve as $\alpha$ decreases to $\gamma _i$, however, remain constant once we have $\alpha \le \gamma _i$.

### Example 2.6

(Fractional stochastic wave equation) Here we consider the fractional stochastic wave equation (4) with mild solution given by (17) with parameters $\alpha \in (1,2)$, $\beta =\kappa =1$, $u_0=U_0$ and $u_1=U_1$, where the operator family $S^{\alpha ,\beta }$ is defined by (18) via its Laplace transform. We use the setting of the previous example for *A*, *F* and *G*; that is, consider the global Lipschitz case. In this case we have $S^{1}(t)=S^2(t)=S^{\alpha ,1}(t)$ with smoothing properties specified by Lemma 3.1

$$\begin{aligned} \Vert A^\xi S^i(t)x\Vert _{H_0}\le Mt^{-\alpha \xi }\Vert x\Vert _{H_0},\quad \Vert A^\xi \dot{S}^i(t)x\Vert _{H_0}\le Mt^{-\alpha \xi -1}\Vert x\Vert _{H_0},\quad {i=1,2,} \end{aligned}$$

for $\xi \in [0,1]$ and $t>0$. Then, Assumption 2.2 (1) is satisfied for any $0<\gamma _1<\frac{1}{2}$ for $s_1(t)=Mt^{\gamma _1-1}$ and Assumption 2.2 (2) is satisfied for any $0<\gamma _2<1$ for $s_2(t)=Mt^{\gamma _2-1}$. The approximating operators in this case become

$$\begin{aligned} S^1_n(t)=S^2_n(t)=\mathcal {P}_nS^{\alpha ,1}(t)=S^{\alpha ,1}(t)\mathcal {P}_n:=S^{\alpha ,1}_n(t). \end{aligned}$$

For $\nu \in [0,1]$, we set $r_1(n)=r_2(n)=\lambda _{n+1}^{-\nu }$. We then have

$$\begin{aligned} \Psi ^1_n(t)=\Psi ^2_n(t)=\lambda _{n+1}^{\nu }(S^{\alpha ,1}(t)-S^{\alpha ,1}_n(t))=\lambda _{n+1}^{\nu }(I-\mathcal {P}_n)S^{\alpha ,1}(t) \end{aligned}$$

with

$$\begin{aligned} \Vert \Psi ^i_n(t)x\Vert _{H_0}\le C\Vert A^\nu S^{\alpha ,1}(t)x\Vert _{H_0}\le C t^{-\alpha \nu }\Vert x\Vert _{H_0},\,i=1,2\text { and }n\in {\mathbb {N}}, \end{aligned}$$

and

$$\begin{aligned} \Vert \dot{\Psi }^i_n(t)x\Vert _{H_0}\le C\Vert A^\nu {\dot{S}}^{\alpha ,1}(t)x\Vert _{H_0}\le C t^{-\alpha \nu -1}\Vert x\Vert _{H_0},\,i=1,2\text { and }n\in {\mathbb {N}}. \end{aligned}$$

Thus, Assumption 2.3 is satisfied with $r_1(n)=\lambda _{n+1}^{-\nu _1}$ with $\nu _1<\frac{\gamma _1}{\alpha }$ and $h_1(t)=Ct^{\gamma _1-1-\alpha \nu _1}$ and $r_2(n)=\lambda _{n+1}^{-\nu _2}$ with $\nu _2<\frac{\gamma _2}{\alpha }$ and $h_2(t)=Ct^{\gamma _2-1-\alpha \nu _2}$. We see here that the rate deteriorates with increasing $\alpha$.

We will often make use of the following results on the Hölder regularity of deterministic and stochastic convolutions.

### Lemma 2.7

Let $Y_1$ and $Y_2$ be real separable Hilbert spaces. Let $T>0$ and suppose that $\Phi \in L^ {p}(\Omega ;L^{\infty }([0,T];Y_1))$ for some $p\in [1,\infty )$. Let $\Psi :[0,T]\rightarrow \mathcal {L}(Y_1,Y_2)$ be a mapping such that the mapping $t\mapsto \Psi (t) x$ is continuously differentiable on (0, *T*) for all $x\in Y_1$. Suppose, moreover, that there exists a function $g\in L ^1 ([0,T];\mathbb {R}^+_0)$ and a constant $\theta \in (0,1)$ such that for all $t\in (0,T)$ we have

$$\begin{aligned} t^\theta \Vert {\dot{\Psi }}(t) x\Vert _{Y_2} + \theta t^{\theta -1} \Vert \Psi (t) x\Vert _{Y_2}&\le g(t)\Vert x\Vert _{Y_1}, \quad \text {for all } x\in Y_1. \end{aligned}$$

Then,

- the convolution
  $$\begin{aligned} (\Psi *\Phi ):t\mapsto \int _{0}^{t}\Psi (t-s)\Phi (s)\,ds \end{aligned}$$
  is well-defined almost surely;
- there is ${\bar{C}}>0$, depending only on $\theta$, such that
  $$\begin{aligned}&\Big \Vert t\mapsto (\Psi *\Phi )(t) \Big \Vert _{L^{p}(\Omega ;C^{1-\theta }([0,T];Y_2))} \\&\qquad \qquad \le {\bar{C}} \Vert g \Vert _{L ^1 ([0,T];\mathbb {R}^+_0)} \left\| \Phi \right\| _{L^ {p}(\Omega ;L^{\infty }([0,T];Y_1))}; \end{aligned}$$
- there is ${\tilde{C}}>0$, depending only on $\theta$ and *T*, such that
  $$\begin{aligned}&\Big \Vert t\mapsto (\Psi *\Phi )(t) \Big \Vert _{L^{p}(\Omega ;C([0,T];Y_2))} \\&\qquad \qquad \le {\tilde{C}} \Vert g \Vert _{L ^1 ([0,T];\mathbb {R}^+_0)} \left\| \Phi \right\| _{L^ {p}(\Omega ;L^{\infty }([0,T];Y_1))}. \end{aligned}$$

### Proof

Note first that, almost surely, the mapping $s\mapsto \Psi (t-s)\Phi (s)\in L^1([0,T]; Y_2)$ and hence $\Psi *\Phi$ is well defined almost surely. In [17, Proposition 3.6] it is shown, that under the assumptions of the theorem, almost surely, there is ${\bar{C}}>0$, depending only on $\theta$, such that

$$\begin{aligned} \Big \Vert t\mapsto (\Psi *\Phi )(t) \Big \Vert _{C^{1-\theta }([0,T];Y_2)}\le {\bar{C}}\Vert g \Vert _{L ^1 ([0,T];\mathbb {R}^+_0)} \left\| \Phi \right\| _{L^{\infty }([0,T];Y_1)}. \end{aligned}$$

The estimate in (b) follows by taking the *p*th power and expected value of both sides of the inequality. Finally the estimate in (c) follows from the estimate in (b) by noting that $(\Psi *\Phi )(0)=0$. $\square$

### Lemma 2.8

Let $Y_1$ and $Y_2$ be real separable Hilbert spaces. Let $T>0$ and suppose that the process $\Phi :\Omega \times [0,T]\rightarrow L_{HS}(H,Y_1)$ is predictable and that $\Phi \in L^{p'}(\Omega ;L^{\infty }([0,T];L_{HS}(H,Y_1)))$ for some $p'\in (2,\infty ]$. Let $\Psi :[0,T]\rightarrow \mathcal {L}(Y_1,Y_2)$ be a mapping such that the mapping $t\mapsto \Psi (t) x$ is continuously differentiable on (0, *T*) for all $x\in Y_1$. Suppose, moreover, that there exists a function $g\in L ^1 ([0,T];\mathbb {R}^+_0)$ and a constant $\theta \in (0,1)$ such that for all $t\in (0,T)$,

$$\begin{aligned} t^\theta \Vert {\dot{\Psi }}(t) x\Vert _{Y_2} + \theta \, t^{\theta -1} \Vert \Psi (t) x\Vert _{Y_2}&\le g(t)\Vert x\Vert _{Y_1}, \quad \text {for all } x\in Y_1. \end{aligned}$$

Then,

- the stochastic convolution process
  $$\begin{aligned} (\Psi \diamond \Phi ):t\mapsto \int _{0}^{t}\Psi (t-s)\Phi (s)\,dW_H(s) \end{aligned}$$
  is well-defined;
- for any $\gamma \in (0,\frac{1}{2}-\theta -\frac{1}{p'})$ with $\theta <\frac{1}{2} -\frac{1}{p'}$ there exists a modification of $\Psi \diamond \Phi$ such that
  $$\begin{aligned}&\Big \Vert t\mapsto (\Psi \diamond \Phi )(t) \Big \Vert _{L^{p'}(\Omega ;C^{\gamma }([0,T];Y_2))} \\&\qquad \qquad \le {\bar{C}} \Vert g \Vert _{L ^1 ([0,T];\mathbb {R}^+_0)} \left\| \Phi \right\| _{L^{p'}(\Omega ;L^{\infty }(0,T;L_{HS}(H,Y_1)))}, \end{aligned}$$
  where ${\bar{C}}$ only depends on $\gamma ,\theta ,p'$ and *T*;
- the modification of $\Psi \diamond \Phi$ from (b) also satisfies
  $$\begin{aligned}&\Big \Vert t\mapsto (\Psi \diamond \Phi )(t) \Big \Vert _{L^{p'}(\Omega ;C([0,T];Y_2))} \\&\qquad \qquad \le {\tilde{C}} \Vert g \Vert _{L ^1 ([0,T];\mathbb {R}^+_0)} \left\| \Phi \right\| _{L^{p'}(\Omega ;L^{\infty }(0,T;L_{HS}(H,Y_1)))}, \end{aligned}$$
  where ${\tilde{C}}$ only depends on $\gamma ,\theta ,p'$ and *T*.

### Proof

Let $$0<\eta <\frac{1}{2}$$. Then

10 $$\begin{aligned}&\sup _{0\le t\le T}\Vert s\mapsto (t-s)^{-\eta }\Phi (s)\Vert _{L^{p'}(\Omega ;L^{2}([0,T];L_{HS}(H,Y_1)))}\nonumber \\&\quad \le C_{\eta ,T} \Vert \Phi \Vert _{L^{p'}(\Omega ;L^{\infty }([0,T];L_{HS}(H,Y_1)))}. \end{aligned}$$

As $Y_1$ is a Hilbert space, the statement in (a) follows from [17, Lemma 3.2] by noting that in this case $L^{2}([0,T];L_{HS}(H,Y_1))\simeq \gamma ([0,T];H,Y_1)$, where the latter denotes the space of gamma radonifying operators from $L^2([0,T];H)\rightarrow Y_1$, see [17, Section 2.2] for further details. Let $\gamma \in (0,\frac{1}{2}-\theta -\frac{1}{p'})$. Then, there is $0<\eta <\frac{1}{2}$ such that $\gamma \in (0,\eta -\theta -\frac{1}{p'})$. Then, by [17, Corollary 3.4], there exists a modification of $\Psi \diamond \Phi$ and a constant *C* depending on $\gamma , \eta , p'$ such that

$$\begin{aligned}&\Big \Vert t\mapsto (\Psi \diamond \Phi )(t) \Big \Vert _{L^{p'}(\Omega ;C^{\gamma }([0,T];Y_2))} \\&\qquad \qquad \le C \Vert g \Vert _{L ^1 ([0,T];\mathbb {R}^+_0)} \sup _{0\le t\le T}\Vert s\mapsto (t-s)^{-\eta }\Phi (s)\Vert _{L^{p'}(\Omega ;L^{2}([0,T];L_{HS}(H,Y_1)))}\\&\qquad \qquad \le {\bar{C}} \Vert g \Vert _{L ^1 ([0,T];\mathbb {R}^+_0)} \Vert \Phi \Vert _{L^{p'}(\Omega ;L^{\infty }([0,T];L_{HS}(H,Y_1)))}, \end{aligned}$$

where ${\bar{C}}$ depends on $\gamma ,p', T$ and $\eta$, with the latter ultimately depending on $\gamma ,\theta$ and $p'$. We used [17, Corollary 3.4] in the first inequality and (10) in the second. Finally the estimate in (c) follows from the estimate in (b) by noting that $(\Psi \diamond \Phi )(0)=0$. $\square$

Next we state a basic existence and uniqueness result.

### Lemma 2.9

Let $p>2$ and let Assumption 2.1, Assumption 2.2, and Assumption 2.3 be satisfied with $0<\gamma _1<\frac{1}{2} -\frac{1}{p}$ and $0<\gamma _2<1$. Then, Eqs. (5) and (6) have unique $H_0$-predictable solutions *U*, respectively $U_n$, in $L^p(\Omega ; L^\infty ([0,T];H_0))$ with

11 $$\begin{aligned} \begin{aligned}&\Vert U\Vert _{L^p(\Omega ; L^\infty ([0,T];H_0))}\le C(1+\Vert X^0\Vert _{L^p(\Omega ; L^\infty ([0,T];H_0))}); \\&\Vert U_n\Vert _{L^p(\Omega ; L^\infty ([0,T];H_0))}\le C(1+\Vert X^0_n\Vert _{L^p(\Omega ; L^\infty ([0,T];H_0))}) , \end{aligned} \end{aligned}$$

for some $C>0$ depending on $\gamma _1,\gamma _2,p$ and *T*.

### Proof

The proof is fairly standard as it uses Banach’s fixed point theorem and therefore we only sketch a proof. Let $T>0$ and set

$$\begin{aligned} X_T:=\{U\in L^p(\Omega ; L^\infty ([0,T];H_0)):\,U\text { is } H_0\text {-predictable}\}. \end{aligned}$$

For $\lambda >0$, later to be chosen appropriately, we endow $X_T$ with the norm

$$\begin{aligned} \Vert U\Vert ^p_\lambda :={\mathbb E}\ {\mathop {\mathrm{ess\ sup}}\limits _{t\in [0,T]}}\ e^{-\lambda p t}\Vert U(t)\Vert _{H_0}^p. \end{aligned}$$

Note, the latter definition is equivalent to the natural norm of $L^p(\Omega ; L^\infty ([0,T];H_0))$. For $U\in X_T$ define the fixed point map

$$\begin{aligned} \mathcal {L}(U)(t)=X^0(t)+\int _{0}^{t}S^2(t-s)F(s,U(s))\,ds+\int _{0}^{t}S^1(t-s)G(s,U(s))\,dW_H(s) \\ =:X^0(t)+\mathcal {L}_2(U)(t)+\mathcal {L}_1(U)(t). \end{aligned}$$

For $\gamma <\min (\frac{1}{2} -\frac{1}{p}-\gamma _1,1 -\gamma _2)$ and for $i=1,2$, we have

12 $$\begin{aligned} \begin{aligned}&\qquad \left\| \mathcal {L}_i(U)(t)\right\| _{L^p(\Omega ; L^\infty ([0,T];H_0))}\\&\le C_T \left\| \mathcal {L}_i(U)(t)\right\| _{L^{p}(\Omega ;C^{\gamma }([0,T];H_0))}{\le } C \Vert s_i\Vert _{L ^1 ([0,T];\mathbb {R}^+_0)}\left( 1{+}\Vert U\Vert _{L^p(\Omega ; L^\infty ([0,T];H_0)}\right) . \end{aligned}\nonumber \\ \end{aligned}$$

In the first inequality above we used the fact that $\mathcal {L}_i(U)(0)=0$. In the second inequality, for $i=1$, we used Lemma 2.7 with $Y_1=H_2$, $Y_2=H_0$ and the linear growth of *F* while, for $i=2$, we used Lemma 2.8 with $Y_1=H_1$, $Y_2=H_0$ and the linear growth of *G*. Hence, the operator $\mathcal {L}$ is a mapping $X_T\rightarrow X_T$. Next we show that $\mathcal {L}$ is a contraction for $\lambda$ large enough. Indeed, if $U,V\in X_T$, then

$$\begin{aligned}&\left\| \mathcal {L}_2(U)(t)-\mathcal {L}_2(V)(t)\right\| ^p_{\lambda } \\&= {\mathbb E}\ {\mathop {\mathrm{ess\ sup}}\limits _{t\in [0,T]}}\ \left\| \int _0^te^{-\lambda (t-s)}S^2(t-s)e^{-\lambda s}\left( F(s,U(s))-F(s,V(s))\right) \,ds\right\| _{H_0}^p \\&\le C_T \left\| t\mapsto \int _0^te^{-\lambda (t-s)}S^2(t-s)e^{-\lambda s}\left( F(s,U(s))-F(s,V(s))\right) \,ds\right\| _{L^{p}(\Omega ;C^{\gamma }([0,T];H_0))}^p\\&\le C_{p,T} \left( \int _0^ T (2+\lambda t) e^{-\lambda t}s_2(t)\, dt\right) ^p\Vert U-V\Vert _{\lambda }^p, \end{aligned}$$

see the estimate of $I_1$ in the proof of Theorem 2.11 for more details. We similarly have that

$$\begin{aligned} \left\| \mathcal {L}_1(U)(t)-\mathcal {L}_1(V)(t)\right\| ^p_{\lambda } \le C_{p,T} \left( \int _0^ T (2+\lambda t) e^{-\lambda t}s_1(t)\, dt\right) ^p\Vert U-V\Vert _{\lambda }^p, \end{aligned}$$

see the estimate of $I_3$ in the proof of Theorem 2.11 for more details. Thus, by choosing $\lambda >0$ large enough, we conclude that there is a constant $M\in (0,1)$ such that $\left\| \mathcal {L}(U)(t)-\mathcal {L}(V)(t)\right\| _{\lambda }\le M\Vert U-V\Vert _\lambda$ when $\mathcal {L}:X_T\rightarrow X_T$ is a contraction. Therefore, by Banach’s fixed point theorem, there exists a unique $U\in X_T$ with $U=\mathcal {L}(U)$. A similar argument shows, by defining the corresponding mapping $\mathcal {L}_n$ in an obvious way, the existence and uniqueness of $U_n$ with the terms $(2+\lambda t) e^{-\lambda t}s_i(t)$ above replaced by $(2+\lambda t) e^{-\lambda t}(s_i(t)+Ch_i(t))$ (c.f., (7) and (8)) yielding a uniform in *n* contraction constant $0<M'<1$ of $\mathcal {L}_n$. The estimate (11) follows from the following simple estimate

$$\begin{aligned} \Vert U\Vert _{\lambda }=\Vert \mathcal {L}(U)\Vert _\lambda \le \Vert \mathcal {L}(U)-\mathcal {L}(0)\Vert _\lambda +\Vert \mathcal {L}(0)\Vert _\lambda \le M\Vert U\Vert _{\lambda }+\Vert X^0\Vert _\lambda +C_{p,T,\lambda }, \end{aligned}$$

where $\lambda$ is large enough so that $0<M<1$ and similarly for $U_n$. $\square$

### Remark 2.10

(Regularity) Observe that under the assumptions of Lemma 2.9, if also $X_0,X_n^0\in L^p(\Omega ;C^\gamma ([0,T];H_0))$ holds for $\gamma <\min (\frac{1}{2} -\frac{1}{p}-\gamma _1,1 -\gamma _2)$, then

$$\begin{aligned} \begin{aligned}&\Vert U\Vert _{ L^p(\Omega ;C^\gamma ([0,T];H_0))}\le C(1+\Vert X^0\Vert _{ L^p(\Omega ;C^\gamma ([0,T];H_0))}+\Vert X^0\Vert _{L^p(\Omega ; L^\infty ([0,T];H_0))}); \\&\Vert U_n\Vert _{ L^p(\Omega ;C^\gamma ([0,T];H_0))}\le C(1+\Vert X^0_n\Vert _{ L^p(\Omega ;C^\gamma ([0,T];H_0))}+\Vert X^0_n\Vert _{L^p(\Omega ; L^\infty ([0,T];H_0))}). \end{aligned} \end{aligned}$$

Indeed, we have that $U=X^0+\mathcal {L}_2(U)+\mathcal {L}_1(U)$ hence the claim for *U* follows from the second inequality in (12) and (11); with an analogous argument showing the claim for $U_n$.

Now we are ready to present our main result.

### Theorem 2.11

Let $p>2$ and let Assumption 2.1, Assumption 2.2, and Assumption 2.3 be satisfied with $0<\gamma _1<\frac{1}{2} -\frac{1}{p}$ and $0<\gamma _2<1$. Suppose that there exists a number $K>0$, such that

$$\begin{aligned} \Vert X_n^0\Vert _{L^p(\Omega ; L^\infty ([0,T];H_0))}<K \quad \text{ for } \text{ all }\quad n\in {\mathbb {N}}. \end{aligned}$$

Let

$$\begin{aligned} {e}(t) := U(t)-{U}_{n}(t)\quad \text{ and }\quad {e}_0(t):= X^0(t)-X_n^0(t),\quad t\in [0,T], \end{aligned}$$

and let $$0<\gamma <\min (\frac{1}{2} -\frac{1}{p}-\gamma _1,1 -\gamma _2)$$.

Then there exists a constant $C>0$, depending on $\gamma ,\gamma _1,\gamma _2$, *p* and *T*, such that for all $n\in \mathbb {N}$ we have

13 $$\begin{aligned} \Vert e\Vert _{L^p(\Omega ;C^\gamma ([0,T];H_0))}\le C\left( \Vert e_0\Vert _{L^p(\Omega ;C^\gamma ([0,T];H_0))} +\Vert e(0)\Vert _{L^p(\Omega ;H_0)}+r_1(n)+r_2(n)\right) , \end{aligned}$$

where the rate functions $r_i$, $i=1,2$, are introduced in Assumption 2.3.

### Proof

Let $t \in [0,T]$ and fix a parameter $\lambda >0$. Then

$$\begin{aligned}&{ e_{\lambda }(t):=e^{-\lambda t}{e}(t) := e^{-\lambda t}(U(t) - U_n(t))} \\&\quad = e^{-\lambda t}X^0(t)- e^{-\lambda t}X^0_n(t)\\&\quad \quad + \int _0^{t} e^{-\lambda (t-s)}{S}^2 (t-s) e^{-\lambda s}[F(s,U(s))-F(s,U_n(s))]\,ds \\&\quad \quad + \int _0^{t}e^{-\lambda (t-s)}[S^2(t-s)-S^2_n (t-s)]e^{-\lambda s}F(s, U_n(s))\,ds \\&\quad \quad + \int _0^{t}e^{-\lambda (t-s)}{S}^1 (t-s)e^{-\lambda s}[G(s,U(s))-G(s,U_n(s))]\,dW_H(s) \\&\quad \quad + \int _0^{t}e^{-\lambda (t-s)}[S^1(t-s)-S^1_n(t-s)]e^{-\lambda s}G(s, U_n(s))\,dW_H(s) \\&:= e^{\lambda }_0(t)+ I_1(t)+I_2(t)+I_3(t)+I_4(t). \end{aligned}$$

Note that, by Remark 2.10, we have that

$$\begin{aligned} \Vert e\Vert _{L^p(\Omega ;C^\gamma ([0,T];H_0))}<\infty \end{aligned}$$

and hence also

$$\begin{aligned} \Vert e_\lambda \Vert _{L^p(\Omega ;C^\gamma ([0,T];H_0))}<\infty . \end{aligned}$$

In the following we estimate term by term.

**Estimate of** $I_1$: To estimate $I_1$, we will use Lemma 2.7 by setting $Y_1=H_2$, $Y_2=H_0$,

$$\begin{aligned}{}[0,T]\ni t\mapsto \Psi (t) := e^{-\lambda t}S^2(t), \end{aligned}$$

and $\Phi (t):= e^{-\lambda t}[F(t,U(t))-F(t,U_n(t))]$. We have that $\Phi \in L^ {p}(\Omega ;L^{\infty }([0,T];H_2))$ since *F* is Lipschitz continuous and $U,U_n\in L^ {p}(\Omega ;L^{\infty }([0,T];H_0))$ by Lemma 2.9. Due to Assumption 2.2-(2), we know that the assumptions of Lemma 2.7 are satisfied with $g(t)=(2+\lambda t )e^{-\lambda t}s_2(t)$ and $\theta =\gamma _2$. Indeed, we have that

$$\begin{aligned} {\dot{\Psi }}(t)x=-\lambda e^{-\lambda t}S^2(t)x+e^{-\lambda t}\dot{S}^2(t)x \end{aligned}$$

Therefore, as $$\gamma _2<1$$,

$$\begin{aligned}&t^{\gamma _2} \Vert {\dot{\Psi }}(t) x\Vert _{H_0} + \gamma _2 t^{\gamma _2-1} \Vert \Psi (t)x\Vert _{H_0}\le t^{\gamma _2} \Vert {\dot{\Psi }}(t) x\Vert _{H_0} + t^{\gamma _2-1} \Vert \Psi (t)x\Vert _{H_0}\\&\quad \le \lambda e^{-\lambda t} t^{\gamma _2}\Vert S^2(t)x\Vert _{H_0}+e^{-\lambda t} t^{\gamma _2}\Vert \dot{S}^2(t)x\Vert _{H_0}+ e^{-\lambda t} t^{\gamma _2-1}\Vert S^2(t)x\Vert _{H_0}\\&\quad \le \lambda t e^{-\lambda t} s_2(t)\Vert x\Vert _{H_2}+e^{-\lambda t} s_2(t)\Vert x\Vert _{H_2} + e^{-\lambda t} s_2(t)\Vert x\Vert _{H_2} \\&\quad =(2+\lambda t)e^{-\lambda t}s_2(t)\Vert x\Vert _{H_2}. \end{aligned}$$

Thus, we can infer by Lemma 2.7

$$\begin{aligned}&\Vert I_1\Vert _{L^p(\Omega ;C^\gamma ([0,T];H_0))} \\&\quad \le C \int _0^ T (2+\lambda t) e^{-\lambda t}s_2(t)\, dt \left( {\mathbb E}\ {\mathop {\mathrm{ess\ sup}}\limits _{t\in [0,T]}}\ e^{-\lambda p t}\Vert F(t,U(t))-F(t,U_n(t))\Vert ^{p}_{H_2} \right) ^ \frac{1}{p}. \end{aligned}$$

The Lipschitz continuity of *F* then gives

14 $$\begin{aligned}&\Vert I_1\Vert _{L^p(\Omega ;C^\gamma ([0,T];H_0))}\nonumber \\&\quad \le C\int _0^ T (2+\lambda t) e^{-\lambda t}s_2(t)\, dt\, \left( {\mathbb E}\ {\mathop {\mathrm{ess\ sup}}\limits _{t\in [0,T]}} \left( e^{-\lambda p t}\Vert e(t)\Vert ^p _{H_0}\right) \right) ^{\frac{1}{p}}\nonumber \\&\quad = C\int _0^ T (2+\lambda t) e^{-\lambda t}s_2(t)\, dt \,\left( {\mathbb E}\left( {\mathop {\mathrm{ess\ sup}}\limits _{t\in [0,T]}} \Vert e_{\lambda }(t)\Vert _{H_0}\right) ^p\right) ^{\frac{1}{p}}\nonumber \\&\quad \le C\int _0^ T (2+\lambda t) e^{-\lambda t}s_2(t)\, dt\, \left( {\mathbb E}\left( {\mathop {\mathrm{ess\ sup}}\limits _{t\in [0,T]}} \left( t^{\gamma }\left\| \frac{e_\lambda (t)-e_{\lambda }(0)}{t^\gamma }\right\| _{H_0}+\Vert e(0)\Vert _{H_0}\right) \right) ^{p}\right) ^{\frac{1}{p}}\nonumber \\&\quad \le C \int _0^ T (2+\lambda t) e^{-\lambda t}s_2(t)\, dt \left( T^\gamma \Vert e_\lambda \Vert _{L^p(\Omega ;C^\gamma ([0,T];H_0))}+\Vert e(0)\Vert _{L^p(\Omega ;H_0)}\right) . \end{aligned}$$

**Estimate of** $I_2$: To estimate $I_2$ we will use again Lemma 2.7 by setting $Y_1=H_2$, $Y_2=H_0$,

$$\begin{aligned}{}[0,T]\ni t\mapsto \Psi (t) :=\frac{1}{r_2(n)} e^{-\lambda t}\left[ S^2(t)-S^2_n(t)\right] = e^{-\lambda t}\Psi _n^2(t), \end{aligned}$$

and $\Phi (t):= e^{-\lambda t}F(t,U_n(t))$. We have that $\Phi \in L^ {p}(\Omega ;L^{\infty }([0,T];H_2))$ since *F* is of linear growth in the second variable, uniformly in $t\in [0,T]$, and since we also have that $U_n\in L^ {p}(\Omega ;L^{\infty }([0,T];H_0))$ by Lemma 2.9. Assumption 2.3-(2) implies that the assumptions of Lemma 2.7 are satisfied for all $n\in {\mathbb {N}}$ with $g(t)=(2+\lambda t )e^{-\lambda t}h_2(t)$ and $\theta =\gamma _2$ as a similar calculation as in the case of $I_1$ shows. Therefore, we can infer from Lemma  2.7 that for all $n\in \mathbb {N}$,

$$\begin{aligned}&\frac{1}{r_2(n)}\Vert I_2\Vert _{L^p(\Omega ;C^\gamma ([0,T];H_0))} \\&\qquad \le C \int _0^ T (2+\lambda t )e^{-\lambda t}h_2(t)\, dt \left( {\mathbb E}\ {\mathop {\mathrm{ess\ sup}}\limits _{t\in [0,T]}}\ e^{-\lambda p t} \Vert F(t, U_n(t))\Vert _{H_2}^ {p}\right) ^ \frac{1}{p}\\&\qquad \le C \int _0^ T h_2(t)\, dt \left( {\mathbb E}\ {\mathop {\mathrm{ess\ sup}}\limits _{t\in [0,T]}}\ \Vert F(t, U_n(t))\Vert _{H_2}^ {p} \right) ^ \frac{1}{p}. \end{aligned}$$

The linear growth of *F* gives for all $n\in \mathbb {N}$

$$\begin{aligned} \frac{1}{r_2(n)}\Vert I_2\Vert _{L^p(\Omega ;C^\gamma ([0,T];H_0))} \le C \int _0^ T h_2(t)\, dt \left( 1+{\mathbb E}\ {\mathop {\mathrm{ess\ sup}}\limits _{t\in [0,T]}}\ \Vert U_n(t)\Vert _{H_0}^ {p} \right) ^ \frac{1}{p}. \end{aligned}$$

**Estimate of** $I_3$: Here, we will apply Lemma 2.8 with $Y_1=H_1$, $Y_2=H_0$,

$$\begin{aligned}{}[0,T]\ni t\mapsto \Psi (t) := e^{-\lambda t}S^1(t), \end{aligned}$$

and $\Phi (t):= e^{-\lambda t}[G(t,U(t))-G(t,U_n(t))]$, $t\in [0,T]$. We have that the process $\Phi$ is predictable as *G* is Lipschitz continuous and $U,U_n$ are predictable by Lemma 2.9 and also that $\Phi \in L^{p}(\Omega ;L^{\infty }([0,T];L_{HS}(H,H_1)))$ as $U,U_n\in L^ {p}(\Omega ;L^{\infty }([0,T];H_0))$ again by Lemma 2.9. Due to Assumption 2.2-(1) the assumptions of Lemma  2.8, with $g(t)=(2+\lambda t )e^{-\lambda t}s_1(t)$ and $\theta =\gamma _1$ are satisfied, as a calculation similar to that in the case of $I_1$ shows. Hence, it follows from Lemma  2.8, that

$$\begin{aligned}&\Vert I_3\Vert _{L^p(\Omega ;C^\gamma ([0,T];H_0))} \\&\quad \le C \int _0^ T (2+\lambda t )e^{-\lambda t}s_1(t)\, dt \left( {\mathbb E}\ {\mathop {\mathrm{ess\ sup}}\limits _{t\in [0,T]}}\ e^{-\lambda p t}\Vert G(t,U(t))-G(t,U_n(t))\Vert _{L_{HS} (H, H_1)}^ {p} \right) ^ \frac{1}{p}. \end{aligned}$$

The Lipschitz continuity of *G* then gives

$$\begin{aligned} \Vert I_3\Vert _{L^p(\Omega ;C^\gamma ([0,T];H_0))} \le C \int _0^ T (2+\lambda t )e^{-\lambda t}s_1(t)\, dt\, \left( {\mathbb E}\ {\mathop {\mathrm{ess\ sup}}\limits _{t\in [0,T]}}\ e^{-\lambda p t}\Vert e(t)\Vert ^ {p}_{H_0} \right) ^ \frac{1}{p}. \end{aligned}$$

By the same calculation used for estimating $I_1$ in (14), where $s_2$ is replaced by $s_1$, we get

$$\begin{aligned}&\Vert I_3\Vert _{L^p(\Omega ;C^\gamma ([0,T];H_0))} \\&\quad \le C \int _0^ T (2+\lambda t) e^{-\lambda t}s_1(t)\, dt \left( T^\gamma \Vert e_\lambda \Vert _{L^p(\Omega ;C^\gamma ([0,T];H_0))}+\Vert e(0)\Vert _{L^p(\Omega ;H_0)}\right) . \end{aligned}$$

**Estimate of** $I_4$: To estimate $I_4$ again we will use Lemma 2.8 by setting $Y_1=H_1$, $Y_2=H_0$,

$$\begin{aligned}{}[0,T]\ni t\mapsto \Psi (t) :=\frac{1}{r_1(n)}e^{-\lambda t} \left[ S^1(t)-S^1_n(t)\right] =e^{-\lambda t}\Psi _n^1(t), \end{aligned}$$

and $\Phi (t):= e^{-\lambda t}G(t,U_n(t))$. The process $\Phi$ is predictable as *G* is Lipschitz continuous and $U_n$ is predictable by Lemma 2.9 and $\Phi \in L^{p}(\Omega ;L^{\infty }([0,T];L_{HS}(H,H_1)))$ as *G* is of linear growth in the second variable, uniformly in [0, *T*], and $U_n\in L^ {p}(\Omega ;L^{\infty }([0,T];H_0))$ by Lemma 2.9. Assumption 2.3-(1) implies that the assumption of Lemma 2.8 are satisfied for all $n\in {\mathbb {N}}$ with $g(t)=(2+\lambda t )e^{-\lambda t}h_1(t)$ and $\theta =\gamma _1$, as a calculation similar to that in the case of $I_1$ shows. Hence, we can infer that for all $n\in \mathbb {N}$,

$$\begin{aligned}&\frac{1}{r_1(n)}\Vert I_4\Vert _{L^p(\Omega ;C^\gamma ([0,T];H_0))} \\&\qquad \le C \int _0^ T(2+\lambda t )e^{-\lambda t}h_1(t)\, dt \left( {\mathbb E}\ {\mathop {\mathrm{ess\ sup}}\limits _{t\in [0,T]}}\ e^{-\lambda p t}\Vert G(t,U_n(t))\Vert _{L_{HS} (H, H_1)}^ { p} \right) ^ \frac{1}{p}\\&\qquad \le C \int _0^ Th_1(t)\, dt \left( {\mathbb E}\ {\mathop {\mathrm{ess\ sup}}\limits _{t\in [0,T]}}\ \Vert G(t,U_n(t))\Vert _{L_{HS} (H, H_1)}^ { p}\right) ^ \frac{1}{p}. \end{aligned}$$

The linear growth of *G* then gives, for all $n\in \mathbb {N}$,

$$\begin{aligned} \frac{1}{r_1(n)}\Vert I_4\Vert _{L^p(\Omega ;C^\gamma ([0,T];H_0))} \le C \int _0^ T h_1(t)\, dt \left( 1+ {\mathbb E}\ {\mathop {\mathrm{ess\ sup}}\limits _{t\in [0,T]}}\ \Vert U_n(t)\Vert _{H_0}^ { p} \right) ^ \frac{1}{p}. \end{aligned}$$

In this way we get

15 $$\begin{aligned}&\Vert e_\lambda \Vert _{L^p(\Omega ;C^\gamma ([0,T];H_0))}\le \Vert e^\lambda _0\Vert _{L^p(\Omega ;C^\gamma ([0,T];H_0)) }\nonumber \\&\quad +C\left( r_1(n)+r_2(n)\right) \left( 1+\Vert U_n\Vert _{L^p(\Omega ; L^\infty ([0,T];H_0))}\right) \nonumber \\&\quad +C \int _0^ T (2+\lambda t) e^{-\lambda t}(s_1(t)+s_2(t))\, dt\,\Big (\Vert e_\lambda \Vert _{L^p(\Omega ;C^\gamma ([0,T];H_0))}+\Vert e(0)\Vert _{L^p(\Omega ;H_0)}\Big ) \end{aligned}$$

Note that, by Lemma 2.9 and by our assumption $\Vert X_n^0\Vert _{L^p(\Omega ; L^\infty ([0,T];H_0))}<K$ for all $n\in {\mathbb {N}}$, there exists a constant $C>0$ such that

$$\begin{aligned} \Vert U_n\Vert _{L^p(\Omega ; L^\infty ([0,T];H_0))}\le C\text { for all } n\in {\mathbb {N}}. \end{aligned}$$

Furthermore, using Lebesgue’s Dominated Convergence Theorem, it follows that

$$\begin{aligned} \int _0^ T (2+\lambda t) e^{-\lambda t}(s_1(t)+s_2(t))\, dt \rightarrow 0 \text { as }\lambda \rightarrow \infty , \end{aligned}$$

and, hence, with $\lambda >0$ large enough, we may absorb the last term on the right hand side of (15) into the left hand side to conclude that

16 $$\begin{aligned} \Vert e_\lambda \Vert _{L^p(\Omega ;C^\gamma ([0,T];H_0))}\le C\left( \Vert e^{\lambda }_0\Vert _{L^p(\Omega ;C^\gamma ([0,T];H_0)) }+ \Vert e(0)\Vert _{L^p(\Omega ;H_0)}+r_1(n)+r_2(n)\right) .\nonumber \\ \end{aligned}$$

Remembering that $e_\lambda (0)= e(0)$ a straightforward calculation shows that

$$\begin{aligned} \Vert e\Vert _{L^p(\Omega ;C^\gamma ([0,T];H_0))} \le C(\lambda ,T)\left( \Vert e_\lambda \Vert _{L^p(\Omega ;C^\gamma ([0,T];H_0))}+\Vert e(0)\Vert _{L^p(\Omega :H_0)}\right) . \end{aligned}$$

A similar calculation implies that, remembering that $e_0(0)=e(0)$,

$$\begin{aligned} \Vert e^{\lambda }_0\Vert _{L^p(\Omega ;C^\gamma ([0,T];H_0))} \le C(\lambda ,T)\left( \Vert e_0\Vert _{L^p(\Omega ;C^\gamma ([0,T];H_0))}+\Vert e(0)\Vert _{L^p(\Omega :H_0)}\right) , \end{aligned}$$

and the proof is complete in view of (16). $\square$

We end this section with the special important case $\gamma =0$.

### Corollary 2.12

Let $p>2$ and let Assumptions 2.1 – 2.3 be satisfied with $0<\gamma _1<\frac{1}{2} -\frac{1}{p}$ and $0<\gamma _2<1$. Suppose that, there exists $K>0$, such that $\Vert X_n^0\Vert _{L^p(\Omega ; L^\infty ([0,T];H_0))}<K$ for all $n\in {\mathbb {N}}$. Let ${e}(t) := U(t)-{U}_{n}(t)$ and ${e}_0(t):= X^0(t)-X^0_n(t)$, $t\in [0,T]$. Then there exists a constant $C>0$, depending on $\gamma _1,\gamma _2$, *p* and *T*, such that, for all $n\in \mathbb {N}$,

$$\begin{aligned} \Vert e\Vert _{L^p(\Omega ;C([0,T];H_0))}\le C\left( \Vert e_0\Vert _{L^p(\Omega ;C([0,T];H_0))} +r_1(n)+r_2(n)\right) , \end{aligned}$$

where the rate functions $r_i$, $i=1,2$, are introduced in Assumption 2.3.

### Proof

In a completely analogous fashion and using the same notation as in the proof of Theorem 2.11 using this time specifically item (c) from Lemma 2.7 and 2.8 one concludes that

$$\begin{aligned} \begin{aligned}&\Vert e_\lambda \Vert _{L^p(\Omega ;C([0,T];H_0))}\le \Vert e^\lambda _0\Vert _{L^p(\Omega ;C([0,T];H_0)) }\\&\quad +C\left( r_1(n)+r_2(n)\right) \left( 1+\Vert U_n\Vert _{L^p(\Omega ; L^\infty ([0,T];H_0))}\right) \\&\quad +C \int _0^ T (2+\lambda t) e^{-\lambda t}(s_1(t)+s_2(t))\, dt\,\Vert e_\lambda \Vert _{L^p(\Omega ;C([0,T];H_0))} . \end{aligned} \end{aligned}$$

Now the proof can be completed the same way as that of Theorem 2.11. $\square$

## Applications

In this section we give two typical instances where our abstract results are applicable.

### Spectral Galerkin approximations of a class of abstract stochastic integral equations

Let $A:D(A)\subset H_0\rightarrow H_0$ be an unbounded, densely defined, self-adjoint, positive definite operator with compact inverse. Let $\lambda _n$ denote the eigenvalues of *A*, arranged in a non-decreasing order, with corresponding orthonormal eigenbasis $(e_n)\subset H_0$. For $\alpha \in (0,2)$, $\beta >1/2$ and $\kappa >0$ we consider the integral equation introduced in [23] given by

17 $$\begin{aligned}&U(t)=S^{\alpha ,1}(t)u_0+S^{\alpha ,2}(t)u_1\nonumber \\&\quad +\int _0^t S^{\alpha ,\kappa }(t-s)F(U(s))\,d s+\int _0^tS^{\alpha ,\beta }(t-s)G(U(s))\,d W_H(s) \end{aligned}$$

where the Laplace transform $\widehat{S^{\alpha ,\beta }}(z)x:=\int _0^\infty e^{-zt}S^{\alpha ,\beta }(t)x\,dt$, $x\in H_0$, $\Re z >0$, of $S^{\alpha ,\beta }$ is given by

18 $$\begin{aligned} \widehat{S^{\alpha ,\beta }}(z)x=z^{\alpha -\beta }(z^{\alpha }+A)^{-1}x \end{aligned}$$

and $u_0,u_1\in L^p(\Omega ;H_0)$ for some $p> 2$ are $\mathcal {F}_0$-measurable. To connect $S^{\alpha ,\beta }$ to the convolution kernels $t\mapsto \frac{1}{\Gamma (\alpha )}t^{\alpha -1}$ and $t\mapsto \frac{1}{\Gamma (\beta )}t^{\beta -1}$ we note that it is shown in [23, Lemma 5.4] that for $\alpha \in (0,2)$, $\beta >0$, and $x\in H_0$ one has

19 $$\begin{aligned} S^{\alpha ,\beta }(t)x=A\int _0^t \frac{(t-s)^{\alpha -1}}{\Gamma (\alpha )} S^{\alpha ,\beta }(s)x\,ds+ \frac{1}{\Gamma (\beta )}t^{\beta -1}x. \end{aligned}$$

Next we provide smoothing estimates for $S^{\alpha ,\beta }$ and its derivative.

#### Lemma 3.1

For $\xi \in [0,1]$, $\alpha \in (0,2)$ and $\beta >0$ the estimates

20 $$\begin{aligned}&\Vert A^\xi S^{\alpha ,\beta }(t)\Vert _{\mathcal {L}(H_0)}\le Mt^{\beta -\alpha \xi -1},\,t>0, \end{aligned}$$

21 $$\begin{aligned}&\Vert A^\xi {\dot{S}}^{\alpha ,\beta }(t)\Vert _{\mathcal {L}(H_0)}\le Mt^{\beta -\alpha \xi -2},\,t>0, \end{aligned}$$

hold for some $M=M(\alpha ,\beta ,\xi )$.

#### Proof

It is shown in [23, Lemma 5.4] that for all $x\in H_0$,

$$\begin{aligned} S^{\alpha ,\beta }(t)x=\frac{1}{2\pi i}\int _{\Gamma _{\rho ,\phi }}e^{zt}z^{\alpha -\beta }(z^\alpha +A)^{-1}x\,dz,\nonumber \\ \end{aligned}$$

where the contour is given by

$$\begin{aligned} \Gamma _{\rho ,\phi }(s)= {\left\{ \begin{array}{ll} (s-\phi +\rho )e^{i\phi }&{}\text { for }s>\phi ,\\ \rho e^{is} &{}\text { for } s\in (-\phi ,\phi ),\\ (-s-\phi +\rho )e^{-i\phi } &{}\text { for }s<-\phi , \end{array}\right. } \end{aligned}$$

where $\rho >0$, $\phi >\frac{\pi }{2}$ and $\alpha \phi <\pi$. Therefore, as $A^{\xi }$ is a closed operator, one has that $S^{\alpha ,\beta }(t)x\in \mathcal {D}(A^\xi )$ and

22 $$\begin{aligned} A^\xi S^{\alpha ,\beta }(t)x=\frac{1}{2\pi i}\int _{\Gamma _{\rho ,\phi }}e^{zt}z^{\alpha -\beta }A^{\xi }(z^\alpha +A)^{-1}x\,dz \end{aligned}$$

provided that

$$\begin{aligned} \int _{\Gamma _{\rho ,\phi }}\left| e^{zt}z^{\alpha -\beta }\right| \left\| A^\xi (z^\alpha +A)^{-1}x\right\| _{H_0}\,|dz|<\infty . \end{aligned}$$

It is shown in the proof of [23, Lemma 5.4] that

23 $$\begin{aligned} \int _{\Gamma _{\rho ,\phi }}\left| e^{zt}z^{\alpha -\beta }\right| \left\| A^\xi (z^\alpha +A)^{-1}x\right\| _{H_0}\,|dz|\le Ct^{\beta -\alpha \xi -1}\Vert x\Vert _{H_0}\int _{\Gamma _{1, \phi }} e^{\Re z} |z|^{-\beta +\alpha \xi }\,|dz|, \end{aligned}$$

and hence (22) holds and

$$\begin{aligned} \Vert A^\xi S^{\alpha ,\beta }(t)\Vert _{\mathcal {L}(H_0)}\le C t^{\beta -\alpha \xi -1},\,t>0. \end{aligned}$$

To show (21) note that, by [23, Lemma 5.4] the function $t\rightarrow S^{\alpha ,\beta }(t)x$ can be extended analytically to a sector in the right half-plane for all $x\in H_0$. In particular, the function $t\rightarrow S^{\alpha ,\beta }(t)x$ is differentiable for $t>0$. Hence, we have

24 $$\begin{aligned} A^{\xi } {\dot{S}}^{\alpha ,\beta }(t)x=\frac{1}{2\pi i}\int _{\Gamma _{\rho ,\phi }}ze^{zt}z^{\alpha -\beta }A^{\xi }(z^\alpha +A)^{-1}x\,dz, \end{aligned}$$

provided that for every $t>0$ there is $\epsilon >0$ and $K=K(t,\epsilon )>0$ such that

$$\begin{aligned} \int _{\Gamma _{\rho ,\phi }}\left| ze^{zt}z^{\alpha -\beta }\right| \left\| A^{\xi }(z^\alpha +A)^{-1}x\right\| _{H_0}\,|dz| <K \end{aligned}$$

for $t\in (t_0-\epsilon ,t_0+\epsilon )$. In a completely analogous fashion as in the case of estimate (23), we get

$$\begin{aligned} \int _{\Gamma _{\rho ,\phi }}\left| ze^{zt}z^{\alpha -\beta }\right| \left\| A^{\xi }(z^\alpha +A)^{-1}x\right\| _{H_0}\,|dz| \\ \le Ct^{\beta -\alpha \xi -2}\Vert x\Vert _{H_0}\int _{\Gamma _{1, \phi }} e^{\Re z} |z|^{-\beta +\alpha \xi +1}\,|dz|, \end{aligned}$$

and thus (24) holds and

$$\begin{aligned} \Vert A^\xi {\dot{S}}^{\alpha ,\beta }(t)\Vert _{\mathcal {L}(H_0)}\le Mt^{\beta -\alpha \xi -2},\,t>0, \end{aligned}$$

which finishes the proof. $\square$

#### Remark 3.2

We would like to point out two crucial points concerning (20) and (21). First, unless $\alpha =\beta =1$, which correspond the heat equation, the estimates do not hold for $\xi >1$. Furthermore, the constant *M* in the estimates blows up as $\alpha \rightarrow 2$ as in this case we must have $\phi \rightarrow \pi /2$ and hence we integrate on a path with infinite line segments approaching the imaginary axis.

#### Assumption 3.3

We assume that

- there exists $0\le \delta _F\le 1$ such that $F: H_0\rightarrow H_{-\delta _F}^A$ is Lipschitz continuous. In particular, there exists a constant $C>0$ with
  $$\begin{aligned} \Vert A^{-\delta _F}(F(x)-F(y))\Vert _{H_0}\le C\, \Vert x-y\Vert _{H_0},\quad x,y\in H_0. \end{aligned}$$
- There exists $0\le \delta _G\le 1$ such that $G: H_0\rightarrow L_{HS}(H,H_{-\delta _G}^A )$ is Lipschitz continuous. In particular, there exists a constant $C>0$ with
  $$\begin{aligned} \Vert A^{-\delta _G}(G(x)-G(y))\Vert _{L_{HS}(H_0,H_0)}\le C\, \Vert x-y\Vert _{H_0},\quad x,y\in H_0{.} \end{aligned}$$

Thus, the spaces $H_1$ and $H_2$ become

$$\begin{aligned} H_1:=H_{-\delta _G}^A \quad \text{ and }\quad H_2:=H_{-\delta _F}^A. \end{aligned}$$

Note next that $A^\xi$ commutes with $S^{\alpha ,\beta }(t)$ for all $t\ge 0$ and all $\xi \in [-1,0]$ using an inversion formula for the Laplace transform (see, for example, [2, Chapter 2.4]) as this property clearly holds for $\widehat{S^{\alpha ,\beta }}(z)$, $\Re z>0$, and $A^\xi \in \mathcal {L}(H_0)$ is this case. Then, for $t>0$, (20) and (21) show that

25 $$\begin{aligned} \begin{aligned}&\Vert S^{\alpha ,\beta }(t)x\Vert _{H_0}\le Mt^{\beta -\alpha \delta _G-1}\Vert x\Vert _{H_1},\quad \Vert {\dot{S}}^{\alpha ,\beta }(t)x\Vert _{H_0}\le Mt^{\beta -\alpha \delta _G-2}\Vert x\Vert _{H_1};\\&\Vert S^{\alpha ,\kappa }(t)x\Vert _{H_0}\le Mt^{\kappa -\alpha \delta _F-1}\Vert x\Vert _{H_2},\quad \Vert {\dot{S}}^{\alpha ,\kappa }(t)x\Vert _{H_0}\le Mt^{\kappa -\alpha \delta _F-2}\Vert x\Vert _{H_2}. \end{aligned} \end{aligned}$$

We also define the approximating operators by

$$\begin{aligned} S^{\alpha ,\beta }_n(t):=S^{\alpha ,\beta }(t)\mathcal {P}_n=\mathcal {P}_nS^{\alpha ,\beta }(t), \end{aligned}$$

where $\mathcal {P}_n$ is defined in (9), and the approximating process by

26 $$\begin{aligned} U_n(t)= & {} S_n^{\alpha ,1}(t)u_0+S_n^{\alpha ,2}(t)u_1+\int _0^t S_n^{\alpha ,\kappa }(t-s)F(U_n(s))\,d s\nonumber \\&\quad +\int _0^tS_n^{\alpha ,\beta }(t-s)G(U_n(s))\,d s. \end{aligned}$$

For $$\nu \ge 0$$, let

$$\begin{aligned} \Psi ^1_n(t):=\lambda _{n+1}^\nu (S^{\alpha ,\beta }(t)-S_n^{\alpha ,\beta }(t))=\lambda _{n+1}^\nu (I-\mathcal {P}_n)S^{\alpha ,\beta }(t) \end{aligned}$$

and

$$\begin{aligned} \Psi ^2_n(t):=\lambda _{n+1}^\nu (S^{\alpha ,\kappa }(t)-S_n^{\alpha ,\kappa }(t))=\lambda _{n+1}^\nu (I-\mathcal {P}_n)S^{\alpha ,\kappa }(t). \end{aligned}$$

For $t>0$, we then have for $\delta _G+\nu \le 1$ and $\delta _F+\nu \le 1$,

27 $$\begin{aligned} \begin{aligned}&\Vert \Psi ^1_n(t)x\Vert _{H_0}\le C \Vert A^\nu S^{\alpha ,\beta }(t)x\Vert _{H_0} \le Ct^{\beta -\alpha (\delta _G+\nu )-1}\Vert x\Vert _{H_1};\\&\Vert \dot{\Psi }^1_n(t)x\Vert _{H_0}\le C \Vert A^\nu {\dot{S}}^{\alpha ,\beta }(t)x\Vert _{H_0} \le C t^{\beta -\alpha (\delta _G+\nu )-2}\Vert x\Vert _{H_1};\\&\Vert \Psi ^2_n(t)x\Vert _{H_0}\le C\Vert A^\nu S^{\alpha ,\kappa }(t)x\Vert _{H_0} \le C t^{\kappa -\alpha (\delta _F+\nu )-1}\Vert x\Vert _{H_2};\\&\Vert \dot{\Psi }^2_n(t)x\Vert _{H_0}\le C \Vert A^\nu {\dot{S}}^{\alpha ,\kappa }(t)x\Vert _{H_0} \le C t^{\kappa -\alpha (\delta _F+\nu )-2}\Vert x\Vert _{H_2}. \end{aligned} \end{aligned}$$

#### Theorem 3.4

Let *U* and $\{U_n:n\in \mathbb {N}\}$ given by (17) and (26), respectively. Let ${e}(t) := U(t)-U_n(t)$ and $e_0(t):=S^{\alpha ,1}(t)u_0-S^{\alpha ,1}_n(t)u_0+S^{\alpha ,2}(t)u_1-S^{\alpha ,2}_n(t)u_1.$

- Let $p>2$, $0< \gamma _1<\frac{1}{2}-\frac{1}{p}$ and $0< \gamma _2<1$ and suppose that $\min (\gamma _1+\beta -\alpha \delta _G-1,\gamma _2+\kappa -\alpha \delta _F-1)>0$. Let $\gamma <\min (\frac{1}{2}-\frac{1}{p}-\gamma _1,1-\gamma _2)$, $\nu _1<\frac{\gamma _1+\beta -\alpha \delta _G-1}{\alpha }$ and $\nu _2<\frac{\gamma _2+\kappa -\alpha \delta _F-1}{\alpha }$. If $\delta _G+\nu _1\le 1$ and $\delta _F+\nu _2\le 1$, then the error estimate
  28 $$\begin{aligned}&\Vert e\Vert _{L^p(\Omega ;C^\gamma ([0,T];H_0))}\le C(T,p, \nu _1,\nu _2,\gamma )\big ( \Vert e_0\Vert _{L^p(\Omega ;C^\gamma ([0,T];H_0))} \nonumber \\&\quad +\Vert e(0)\Vert _{L^p(\Omega ;H_0)}+\lambda _{n+1}^{-\nu _1}+\lambda _{n+1}^{-\nu _2}\big ) \end{aligned}$$
  holds. Set $\nu :=\min (\nu _1,\nu _2)$ and suppose that $\nu +\frac{\gamma }{\alpha }\le 1$. If $u_0\in L^p(\Omega ;\mathcal {D}(A^{\nu +\frac{\gamma }{\alpha }}))$ and $u_1\in L^p(\Omega ;\mathcal {D}(A^{\max (0,\nu +\frac{\gamma -1}{\alpha })}))$, then
  $$\begin{aligned} \Vert e\Vert _{L^p(\Omega ;C^\gamma ([0,T];H_0))}\le C(T,p, \nu _1,\nu _2,\gamma , u_0,u_1)\lambda _{n+1}^{-\nu }. \end{aligned}$$
- Let $p>2$ and suppose that $\min (\frac{1}{2}-\frac{1}{p}+\beta -\alpha \delta _G-1,\kappa -\alpha \delta _F)>0$. For $\nu _1<\frac{\frac{1}{2}-\frac{1}{p}+\beta -\alpha \delta _G-1}{\alpha }$ and $\nu _2<\frac{\kappa -\alpha \delta _F}{\alpha }$, if $\delta _G+\nu _1\le 1$ and $\delta _F+\nu _2\le 1$, then the error estimate
  29 $$\begin{aligned} \Vert e\Vert _{L^p(\Omega ;C([0,T];H_0))}\le C(T,p, \nu _1,\nu _2)\big ( \Vert e_0\Vert _{L^p(\Omega ;C([0,T];H_0))} +\lambda _{n+1}^{-\nu _1}+\lambda _{n+1}^{-\nu _2}\big )\nonumber \\ \end{aligned}$$
  holds. Setting $\nu :=\min (\nu _1,\nu _2)$, and assuming that $u_0\in L^p(\Omega ;\mathcal {D}(A^{\nu }))$ and $u_1\in L^p(\Omega ;\mathcal {D}(A^{\max (0,\nu -\frac{1}{\alpha })}))$ the error estimate
  $$\begin{aligned} \Vert e\Vert _{L^p(\Omega ;C([0,T];H_0))}\le C(T,p, \nu _1,\nu _2, u_0,u_1)\lambda _{n+1}^{-\nu } \end{aligned}$$
  holds.

#### Proof

Estimate (28) follows from Theorem 2.11 using Assumption 3.3 and estimates (25) and (27). To estimate the initial terms first note that as $S^{\alpha ,1}(0)=I$ and $S^{\alpha ,2}(0)=0$ it follows that

$$\begin{aligned} \Vert e(0)\Vert _{L^p(\Omega ;H_0)}=\Vert u_0-\mathcal {P}_nu_0\Vert _{L^p(\Omega ;H_0)}\le C \lambda _{n+1}^{-\nu }\Vert A^\nu u_0\Vert _{L^p(\Omega ;H_0)}. \end{aligned}$$

It is shown in [23, Lemma 5.4] that if $x\in \mathcal {D}(A^{\nu +\frac{\gamma }{\alpha }})$ with $\nu +\frac{\gamma }{\alpha }\le 1$, then the function $v(t):= S^{\alpha ,1}(t)x-x$ admits a fractional derivative of order $\gamma$ defined by

$$\begin{aligned} D^{\gamma }_tv(t)=\frac{1}{\Gamma (1-\gamma )}\frac{d}{dt}\int _0^t(t-s)^{-\gamma }v(s)\,d s,\,\gamma \in (0,1), \end{aligned}$$

in $$\mathcal {D}(A^\nu )$$ and

$$\begin{aligned} \Vert A^\nu D^{\gamma }_tv(t)\Vert _{H_0}\le M\Vert A^{\nu +\frac{\gamma }{\alpha }}x\Vert _{H_0}, \,t\in (0,T]. \end{aligned}$$

A straightforward calculation shows that (see also, [57, Vol. II, p. 138], [15])

$$\begin{aligned} \Vert A^\nu v(\cdot )\Vert _{C^\gamma ([0,T];H_0)} \le C \Vert A^\nu D^{\gamma }_tv(\cdot )\Vert _{L^{\infty }([0,T];H_0)}. \end{aligned}$$

Therefore,

$$\begin{aligned}&\Vert S^{\alpha ,1}(\cdot )u_0-S^{\alpha ,1}_n(\cdot )u_0\Vert _{L^p(\Omega ;C^\gamma ([0,T];H_0))}\\&=\Vert S^{\alpha ,1}(\cdot )(I-\mathcal {P}_n)u_0\Vert _{L^p(\Omega ;C^\gamma ([0,T];H_0))}\\&\le \lambda _{n+1}^{-\nu }\Vert A^\nu S^{\alpha ,1}(\cdot )u_0\Vert _{L^p(\Omega ;C^\gamma ([0,T];H_0))}\\&=\lambda _{n+1}^{-\nu }\Vert A^\nu (S^{\alpha ,1}(\cdot )u_0-u_0)\Vert _{L^p(\Omega ;C^\gamma ([0,T];H_0))}\\&\le C \lambda _{n+1}^{-\nu }\Vert A^\nu D^{\gamma }_t (S^{\alpha ,1}(\cdot )u_0-u_0)\Vert _{L^p(\Omega ;L^{\infty }([0,T];H_0))}\\&\le C \lambda _{n+1}^{-\nu } \Vert A^{\nu +\frac{\gamma }{\alpha }} u_0\Vert _{L^p(\Omega ;H_0)}. \end{aligned}$$

Here, for the equality in the second row of the calculation, we used the fact that the $C^\gamma ([0,T];H_0))$-seminorm of a function $f:[0,T]\rightarrow H_0$ does not change by adding a constant to *f*. Similarly, it is also shown in [23, Lemma 5.4] that if $x\in \mathcal {D}(A^{\max (0,\nu +\frac{\gamma -1}{\alpha })})$, then the function $v(t):= S^{\alpha ,2}(t)x$ admits a fractional derivative in $\mathcal {D}(A^\nu )$ and

$$\begin{aligned} \Vert A^\nu D^{\gamma }_tv(t)\Vert _{H_0}\le M\Vert A^{\max (0,\nu +\frac{\gamma -1}{\alpha })}x\Vert _{H_0}, \,t\in (0,T]. \end{aligned}$$

Then, similarly as above we conclude that

$$\begin{aligned} \Vert S^{\alpha ,2}(\cdot )u_1-S^{\alpha ,2}_n(\cdot )u_1\Vert _{L^p(\Omega ;C^\gamma ([0,T];H_0))}\le C \lambda _{n+1}^{-\nu } \Vert A^{\max (0,\nu +\frac{\gamma -1}{\alpha })} u_1\Vert _{L^p(\Omega ;H_0)} \end{aligned}$$

and the proof of (a) is complete. To show the claims in (b) first note that estimate (29) follows from Corollary 2.12 by choosing $\gamma _1$ sufficiently close to $\frac{1}{2}-\frac{1}{p}$ and $\gamma _2$ sufficiently close to 1 together with Assumption 3.3 and estimates (25) and (27). To estimate the initial terms first note that, by (20) with $\xi =0$ and $\beta =1$, using also the fact that $S^{\alpha ,1}$ and $A^{-\nu }$ commutes for $\nu \in [0,1]$,

$$\begin{aligned}&\Vert S^{\alpha ,1}(t)u_0-S^{\alpha ,1}_n(t)u_0\Vert _{H_0}=\Vert (I-\mathcal {P}_n)S^{\alpha ,1}(t)u_0\Vert _{H_0}\le C \lambda _{n+1}^{-\nu }\Vert A^\nu S^{\alpha ,1}(t)u_0\Vert _{H_0}\\&\quad =C \lambda _{n+1}^{-\nu }\Vert A^\nu S^{\alpha ,1}(t)A^{-\nu }A^\nu u_0\Vert _{H_0}=C \lambda _{n+1}^{-\nu }\Vert S^{\alpha ,1}(t)A^\nu u_0\Vert _{H_0}\le C \lambda _{n+1}^{-\nu } \Vert A^\nu u_0\Vert _{H_0}, \end{aligned}$$

for all $t\ge 0$. Therefore,

$$\begin{aligned} \Vert S^{\alpha ,1}(\cdot )u_0-S^{\alpha ,1}_n(\cdot )u_0\Vert _{L^p(\Omega ;C([0,T];H_0))}\le C \lambda _{n+1}^{-\nu } \Vert A^{\nu } u_0\Vert _{L^p(\Omega ;H_0)}. \end{aligned}$$

For the second initial term, let first $\nu \le \frac{1}{\alpha }$. Then, by (20) with $\xi =\nu$ and $\beta =2$,

$$\begin{aligned}&\Vert S^{\alpha ,2}(t)u_1-S^{\alpha ,2}_n(t)u_1\Vert _{H_0}=\Vert (I-\mathcal {P}_n)S^{\alpha ,2}(t)u_1\Vert _{H_0}\le C \lambda _{n+1}^{-\nu }\Vert A^\nu S^{\alpha ,2}(t)u_1\Vert _{H_0}\\&\quad =C t^{1-\alpha \nu }\lambda _{n+1}^{-\nu }\Vert u_1\Vert _{H_0},\, t\ge 0. \end{aligned}$$

On the other hand, if $1\ge \nu > \frac{1}{\alpha }$, then by (20) with $\xi =\frac{1}{\alpha }$ and $\beta =2$,

$$\begin{aligned} \Vert S^{\alpha ,2}(t)u_1-S^{\alpha ,2}_n(t)u_1\Vert _{H_0}=\Vert (I-\mathcal {P}_n)S^{\alpha ,2}(t)u_1\Vert _{H_0}\le C \lambda _{n+1}^{-\nu }\Vert A^\nu S^{\alpha ,2}(t)u_1\Vert _{H_0}\\ =C \lambda _{n+1}^{-\nu }\Vert A^{\frac{1}{\alpha }} S^{\alpha ,2}(t)A^{\nu -\frac{1}{\alpha }}u_1\Vert _{H_0}\le C \lambda _{n+1}^{-\nu }\Vert A^{\nu -\frac{1}{\alpha }}u_1\Vert _{H_0},\,t\ge 0. \end{aligned}$$

In summary, if $u_1\in L^p(\Omega ;\mathcal {D}(A^{\max (0,\nu -\frac{1}{\alpha })}))$, then

$$\begin{aligned} \Vert S^{\alpha ,2}(\cdot )u_1-S^{\alpha ,2}_n(\cdot )u_1\Vert _{L^p(\Omega ;C^\gamma ([0,T];H_0))}\le C \lambda _{n+1}^{-\nu } \Vert A^{\max (0,\nu -\frac{1}{\alpha })} u_1\Vert _{L^p(\Omega ;H_0)}, \end{aligned}$$

which finishes the proof of item (b) and hence that of the theorem. $\square$

#### Remark 3.5

In general, we may observe that larger values of the parameters $\beta$ and $\kappa$ allow for higher rates of convergence in Theorem 3.4 as larger values of these parameters correspond to better time-regularity of the stochastic, respectively, deterministic feedback. Note also, that the operator family $S^{\alpha ,2}$, which is $S^{\alpha ,\beta }$ with $\beta =2$ has a stronger smoothing effect than $S^{\alpha ,1}$, which is $S^{\alpha ,\beta }$ with $\beta =1$, as Lemma 3.1 shows. This explains why the regularity requirement in Theorem 3.4 is stricter on $u_0$ than that on $u_1$ for the same rate of convergence.

#### Example 3.6

(Stochastic heat equation) The stochastic heat equation corresponds to parameters $\alpha =\beta =\kappa =1$ and $u_1=0$. In this case, Theorem 3.4, yields

$$\begin{aligned} \Vert e\Vert _{L^p(\Omega ;C^\gamma ([0,T];H_0))}\le C\lambda _{n+1}^{-\nu }, \end{aligned}$$

for $\gamma <\min (\frac{1}{2}-\frac{1}{p}-\gamma _1,1-\gamma _2)$ and $\nu =\min (\nu _1,\nu _2)$ where $\nu _1<\gamma _1 -\delta _G$ and $\nu _2<\gamma _2 -\delta _F$, provided that $u_0\in L^p(\Omega ;\mathcal {D}(A^{\nu +\gamma }))$. Furthermore,

$$\begin{aligned} \Vert e\Vert _{L^p(\Omega ;C([0,T];H_0))}\le C\lambda _{n+1}^{-\nu } \end{aligned}$$

for $\nu =\min (\nu _1,\nu _2)$ where $\nu _1< \frac{1}{2}-\frac{1}{p}-\delta _G$ and $\nu _2<1 -\delta _F$, provided that $u_0\in L^p(\Omega ;\mathcal {D}(A^{\nu }))$. This is consistent with [16, Proposition 3.1].

#### Example 3.7

(Fractional stochastic heat equation) The simplest fractional stochastic heat equation, considered also in Example 2.5 in the standard global Lipschitz setting, corresponds to parameters $\beta =\kappa =1$, $\alpha \in (0,1)$ and $u_1=0$. In this case, Theorem 3.4, yields

$$\begin{aligned} \Vert e\Vert _{L^p(\Omega ;C^\gamma ([0,T];H_0))}\le C\lambda _{n+1}^{-\nu }, \end{aligned}$$

for $\gamma <\min (\frac{1}{2}-\frac{1}{p}-\gamma _1,1-\gamma _2)$ and $\nu =\min (\nu _1,\nu _2)$ where $\nu _1<\frac{\gamma _1}{\alpha } -\delta _G$ and $\nu _2<\frac{\gamma _2}{\alpha } -\delta _F$, provided that $u_0\in L^p(\Omega ;\mathcal {D}(A^{\nu +\frac{\gamma }{\alpha }}))$ and $\max (\nu _1+\delta _G,\nu _2+\delta _F)\le 1$ holds. Furthermore,

$$\begin{aligned} \Vert e\Vert _{L^p(\Omega ;C([0,T];H_0))}\le C\lambda _{n+1}^{-\nu } \end{aligned}$$

for $\nu =\min (\nu _1,\nu _2)$ where $\nu _1< \frac{\frac{1}{2}-\frac{1}{p}}{\alpha }-\delta _G$ and $\nu _2<\frac{1}{\alpha } -\delta _F$, provided that $u_0\in L^p(\Omega ;\mathcal {D}(A^{\nu }))$ and $\max (\nu _1+\delta _G,\nu _2+\delta _F)\le 1$ holds. As discussed in Example 2.5, the rate only improves with decreasing $\alpha$ as long as $\max (\nu _1+\delta _G,\nu _2+\delta _F)\le 1$ holds and stays the same when $\alpha$ decreases further. For example, if $\delta _F=\delta _G=0$, then

$$\begin{aligned} \Vert e\Vert _{L^p(\Omega ;C([0,T];H_0))}\le C\lambda _{n+1}^{-\nu } \end{aligned}$$

for $$\nu <\min (\frac{\frac{1}{2}-\frac{1}{p}}{\alpha },1)$$.

#### Example 3.8

(Fractional stochastic wave equation) The simplest fractional stochastic wave equation, considered also in Example 2.6 in the standard global Lipschitz setting, corresponds to parameters $\beta =\kappa =1$, $\alpha \in (1,2)$. In this case, Theorem 3.4, yields

$$\begin{aligned} \Vert e\Vert _{L^p(\Omega ;C^\gamma ([0,T];H_0))}\le C\lambda _{n+1}^{-\nu }, \end{aligned}$$

for $\gamma <\min (\frac{1}{2}-\frac{1}{p}-\gamma _1,1-\gamma _2)$ and $\nu =\min (\nu _1,\nu _2)$ where $\nu _1<\frac{\gamma _1}{\alpha } -\delta _G$ and $\nu _2<\frac{\gamma _2}{\alpha } -\delta _F$, provided that $u_0\in L^p(\Omega ;\mathcal {D}(A^{\nu +\frac{\gamma }{\alpha }}))$ and $u_1\in L^p(\Omega ;\mathcal {D}(A^{\max (0,\nu +\frac{\gamma -1}{\alpha })}))$. Furthermore,

$$\begin{aligned} \Vert e\Vert _{L^p(\Omega ;C([0,T];H_0))}\le C\lambda _{n+1}^{-\nu } \end{aligned}$$

for $\nu =\min (\nu _1,\nu _2)$ where $\nu _1< \frac{\frac{1}{2}-\frac{1}{p}}{\alpha }-\delta _G$ and $\nu _2<\frac{1}{\alpha } -\delta _F$, provided that $u_0\in L^p(\Omega ;\mathcal {D}(A^{\nu }))$, $u_1\in L^p(\Omega ;\mathcal {D}(A^{\max (0,\nu -\frac{1}{\alpha })}))$. We see that the rates deteriorate with increasing $\alpha$.

### Finite element approximation of a stochastic fractional wave equation

In this section we give a more concrete example to demonstrate how the abstract framework can be used for finite element approximation. Let $\mathcal {D}\subset {\mathbb R}^d$ be a bounded convex polygonal domain and let $A=-\Delta$ be the Dirichlet Laplacian in $(H_0,\Vert \cdot \Vert _{H_0}):=(L^2(\mathcal {D}),\Vert \cdot \Vert _{L^2(\mathcal {D})})$ with domain $\mathcal {D}(A)=H^2(\mathcal {D})\cap H^1_0(\mathcal {D})$. Let $p>2$ and consider a fractional stochastic wave equation [13, 38] given by

30 $$\begin{aligned} \left\{ \begin{aligned} dU(t)+A\int _0^tb(t-s)U(s)\,ds\,dt&=F(U(s))dt+R(U(s))Q^\frac{1}{2}d W_H(t),\,t>0; \\ U(0)&=U_0, \end{aligned} \right. \nonumber \\ \end{aligned}$$

where $W_H$ is a cylindrical Wiener process in $H=H_0$ and $Q:H\rightarrow H$ is a linear, symmetric, positive semidefinite, trace class operator on *H* with an orthonormal basis of eigenfunctions $\{e_k:k\in \mathbb {N}\}$ with $\Vert e_k\Vert _{L^\infty (\mathcal {D})}\le M$ for all $k=1,2,\dots$. The initial data $U_0\in L^p(\Omega ;H_0)$ for some $p>2$ is assumed to be $\mathcal {F}_0$-measurable. The kernel *b* is the Riesz kernel given by

$$\begin{aligned} b(t)=\frac{t^{\alpha -1}}{\Gamma (\alpha )},\quad \alpha \in (0,1). \end{aligned}$$

For $u\in H_0$ define $[F(u)](r):=f(u(r))$ with $f:{\mathbb R}\rightarrow {\mathbb R}$ being a globally Lipschitz continuous function while $[R(u)v](r):=g(u(r))v(r)$ with $g:{\mathbb R}\rightarrow {\mathbb R}$ being a globally Lipschitz continuous function. Then we may take $H_0=H_1=H_2=H$ and a straightforward calculation yields that *F* and $G(u)v:=R(u)Q^\frac{1}{2} v$ satisfy Assumption 2.1. The mild solution of (30) is given by the variation of constants formula

31 $$\begin{aligned} U(t)=S(t)U_0+\int _0^tS(t-s)F(U(s))\, ds+\int _0^tS(t-s)G(U(s))\,d W_H(s). \end{aligned}$$

Here the resolvent family $\{S(t)\}_{t\ge 0}$ is a strongly continuous family of bounded linear operators on $H_0$, which is strongly differentiable on $(0,\infty )$ such that the function $t\mapsto S(t)x$ is the unique solution of

32 $$\begin{aligned} {\dot{u}}(t)+A\int _0^t b(t-s)u(s)\,\, d s=0,\,t>0;\,u(0)=x, \end{aligned}$$

see [52, Corollary 1.2].

#### Remark 3.9

In connection with Subsection 3.1, in particular (19), by integrating (32) from 0 to *t*, one sees that in fact $S(t)=S^{\alpha +1,1}(t)$.

The resolvent family *S* has the following smoothing properties [47]:

33 $$\begin{aligned}&\Vert A^\mu S(t)\Vert _{\mathcal {L}(H_0)}\le Ct^{-(\alpha +1)\mu } ,\quad \mu \in [0,1],\,t>0;\nonumber \\&\Vert A^\mu {\dot{S}}(t)\Vert _{\mathcal {L}(H_0)}\le Ct^{-(\alpha +1)\mu -1} ,\quad \mu \in [-1,1],\,t>0. \end{aligned}$$

For spatial approximation of (30) we consider a standard continuous finite element method. Let $\{\mathcal {T}_h\}_{0<h<1}$ denote a family of triangulations of $\mathcal {D}$, with mesh size $h>0$ and consider finite element spaces $\{ V_h \}_{0<h<1}$, where $V_h\subset H^1_0(\mathcal {D})$ consists of continuous piecewise linear functions vanishing at the boundary of $\mathcal {D}$. We introduce the "discrete Laplacian" (see, for example, [55, page 10])

$$\begin{aligned} A_{h}:V_h\rightarrow V_h, \quad (A_{h} \psi , \chi )_{H_0} = ( \nabla \psi ,\nabla \chi )_{H_0},\quad \psi ,\chi \in V_h, \end{aligned}$$

where $(\cdot ,\cdot )_{H_0}$ denotes the inner product of $H_0$, and the orthogonal projection

$$\begin{aligned} P_{h}: H_0 \rightarrow V_h,\quad (P_{h} f, \chi )_{H_0} = ( f,\chi )_{H_0},\quad \chi \in V_h. \end{aligned}$$

We consider the approximated problem

$$\begin{aligned} \left\{ \begin{aligned} dU_h(t)+A_h\int _0^tb(t-s)U_h(s)\,ds\,dt&=P_hF(U_h(s))dt+P_hG(U_h(s))d W_H(t); \\ U_h(0)&=P_hU_0, \end{aligned} \right. \end{aligned}$$

with mild solution given by

34 $$\begin{aligned} U_h(t)= & {} S_h(t)P_hU_0+\int _0^tS_h(t-s)P_hF(U_h(s))\, ds\nonumber \\&\quad +\int _0^tS_h(t-s)P_hG(U_h(s))\,d W_H(s). \end{aligned}$$

Similarly to the resolvent family $\{S(t)\}_{t\ge 0}$, the resolvent family $\{S_h(t)\}_{t\ge 0}$ is a strongly continuous family of bounded linear operators on $V_h$, which is strongly differentiable on $(0,\infty )$ such that for $\chi \in V_h$ the $V_h$-valued function $t\mapsto S_h(t)\chi$ is the unique solution of

35 $$\begin{aligned} {\dot{u}}_h(t)+\int _0^t b(t-s)A_hu_h(s)\,\, d s=0,\,t>0;\,u_h(0)=\chi . \end{aligned}$$

Let $E_h(t):=S(t)-S_h(t)P_h$ denote the deterministic error operator. We have the following error bounds.

#### Proposition 3.10

Let $\epsilon >0$ and $T>0$. Then, the error estimates

36 $$\begin{aligned}&\Vert E_h(t)x\Vert _{H_0}\le C_\epsilon h^\beta \Vert A^{\beta (\frac{1+\epsilon }{2})}x\Vert _{H_0},\quad \beta \in [0,2],\, x\in \mathcal {D}(A^{\beta (\frac{1+\epsilon }{2})}),\,t\in [0,T]; \end{aligned}$$

37 $$\begin{aligned}&\Vert E_h(t)\Vert _{\mathcal {L}(H_0)}\le Ch^{2\beta }t^{-\beta (\alpha +1)},\quad \beta \in [0,1],\,t\in (0,T]; \end{aligned}$$

38 $$\begin{aligned}&\Vert {\dot{E}}_h(t)\Vert _{\mathcal {L}(H_0)} \le Ch^{2\beta }t^{-\beta (\alpha +1)-1},\quad \beta \in [0,1],\,t\in (0,T], \end{aligned}$$

hold for $0<h< 1$ and $C,C_\epsilon >0$.

#### Proof

The error bound (36) is shown in [40, Proposition 3.3]. The error estimate (37) for $\beta =1$ is proved in [46, Theorem 2.1] while for $\beta \in [0,1)$ it follows immediately using also the stability estimate $\Vert E_h(t)\Vert \le C$; the latter is a consequence of (36) with $\beta =0$. Thus, we have to prove (38). It is shown in [46, Eq. (2.2)] that the Laplace transform ${\widehat{E}}(z)$ of $E_h$ satisfies the error estimate

39 $$\begin{aligned} \Vert {\widehat{E}}(z)\Vert _{\mathcal {L}(H_0)}\le C h^2 |z|^\alpha \end{aligned}$$

in a symmetric sectorial region containing the right half-plane. We write

$$\begin{aligned} E_h(t)=S(t)-S_h(t)P_h=S(t)P_h-S_h(t)P_h+S(t)(I-P_h):=E^1_h(t)+E^2_h(t). \end{aligned}$$

Let $x\in \mathcal {D}(A)$. Then, it follows that $t\mapsto E^1_h(t)x$ is continuously differentiable on $[0,\infty )$, $E^1_h(0)x=0$ and

$$\begin{aligned} \Vert {\dot{E}}^1_h(t)x\Vert _{H_0}\le Ct^{\alpha }(\Vert Ax\Vert _{H_0}+\Vert A_hP_hx\Vert _{H_0}). \end{aligned}$$

Hence $t\mapsto {\dot{E}}^1_h(t)x$ is Laplace transformable and

40 $$\begin{aligned} \widehat{{\dot{E}}^1_h}(z)x=z\widehat{E^1_h}(z)x=z{\widehat{E}}(z)P_hx. \end{aligned}$$

Let $\theta \in (\frac{\pi }{2},\frac{\pi }{1+\alpha })$ be fixed and let $\Gamma :=\{z:\, |\arg (z)|=\theta \}$ denote the curve with $\text {Im}\,z$ running from $-\infty$ to $\infty$. Then, using (39) and (40), we get

$$\begin{aligned}&\Vert {\dot{E}}^1_h(t)x\Vert _{H_0}=\left\| \frac{1}{2\pi i}\int _\Gamma e^{tz}z{\widehat{E}}(z)P_hx\,dz\right\| _{H_0} \\&\le Ch^2 \int _\Gamma |z|^{\alpha +1} e^{-ct|z|}\, |dz|\Vert P_hx\Vert _{H_0}\le Ch^2 \int _0^\infty s^{\alpha +1}e^{-cst}\,ds\Vert P_h\Vert _{\mathcal {L}(H_0)}\Vert x\Vert _{H_0}\\&\le Ch^2 \int _0^\infty \left( \frac{r}{t}\right) ^{\alpha +1}e^{-cr}\,\frac{dr}{t}\Vert x\Vert _{H_0} \le C h^2 t^{-(\alpha +1)-1}\Vert x\Vert _{H_0},\,t>0. \end{aligned}$$

Therefore, as $\mathcal {D}(A)$ is dense in $H_0$, we conclude that

$$\begin{aligned} \Vert {\dot{E}}^1_h(t)\Vert _{\mathcal {L}(H_0)}\le C h^2 t^{-(\alpha +1)-1},\,t>0. \end{aligned}$$

To bound ${\dot{E}}^2_h(t)$ recall that $\Vert (I-P_h)x\Vert _{H_0}\le Ch^2\Vert Ax\Vert _{H_0}$. Hence, using the smoothing property (33) and the self-adjointness of $P_h$ and ${\dot{S}}$, we get

$$\begin{aligned}&\Vert {\dot{E}}^2_h(t)\Vert _{\mathcal {L}(H_0)}=\Vert {\dot{S}}(t)(I-P_h)\Vert _{\mathcal {L}(H_0)}=\Vert [{\dot{S}}(t)(I-P_h)]^*\Vert _{\mathcal {L}(H_0)}\\&\quad =\Vert (I-P_h)^*{\dot{S}}(t)^*\Vert _{\mathcal {L}(H_0)} =\Vert (I-P_h){\dot{S}}(t)\Vert _{\mathcal {L}(H_0)}\le Ch^2\Vert A{\dot{S}}(t)\Vert _{\mathcal {L}(H_0)}\\&\quad \le C h^2 t^{-(\alpha +1)-1}, \end{aligned}$$

where $L^*$ denotes the adjoint of an operator $L\in \mathcal {L}(H_0)$. Thus, in summary,

$$\begin{aligned} \Vert {\dot{E}}_h(t)\Vert _{\mathcal {L}(H_0)}\le \Vert {\dot{E}}^1_h(t)\Vert _{\mathcal {L}(H_0)}+\Vert {\dot{E}}^2_h(t)\Vert _{\mathcal {L}(H_0)}\le C h^2 t^{-(\alpha +1)-1},\,t>0, \end{aligned}$$

which is (38) for $\beta =1$. Then, it follows that to show (38) for $\beta \in [0,1]$ it is enough to prove that

$$\begin{aligned} \Vert {\dot{E}}_h(t)\Vert _{\mathcal {L}(H_0)}\le Ct^{-1},\, t>0. \end{aligned}$$

As, by (33), $\Vert {\dot{S}}(t)\Vert _{\mathcal {L}(H_0)}\le Ct^{-1}$, $t>0$, we only need to prove that

41 $$\begin{aligned} \Vert {\dot{S}}_h(t)P_h\Vert _{\mathcal {L}(H_0)}\le Ct^{-1},\,t>0. \end{aligned}$$

It is well-known, see, for example [55, Chapter 6] that the uniform resolvent estimate

42 $$\begin{aligned} \Vert (zI+A_h)^{-1}P_h\Vert _{\mathcal {L}(H_0)}\le \frac{M_\omega }{|z|} \end{aligned}$$

holds in any sector $\Sigma _\omega =\{z:\, |\arg z|<\omega \}\setminus \{0\}$, $\omega \in (0,\pi ).$ A simple calculation shows that

$$\begin{aligned} {\widehat{S_h}}(z)P_h=z^{\alpha }(z^{1+\alpha }I+A_h)^{-1}P_h. \end{aligned}$$

Note that

$$\begin{aligned} \Vert {\dot{S}}_h(t)P_hx\Vert _{H_0}\le Ct^{\alpha }\Vert A_h\Vert _{\mathcal {L}(H_0)}\Vert x\Vert _{H_0} \end{aligned}$$

and thus $t\rightarrow {\dot{S}}_h(t)P_h$ is Laplace transformable and

$$\begin{aligned} \widehat{{\dot{S}}_h}(z)P_h=z^{1+\alpha }(z^{1+\alpha }I+A_h)^{-1}P_h-P_h{,} \end{aligned}$$

for all $z\in \Sigma _\omega$ with $\omega <\pi /(1+\alpha )$. Using (42) it follows that

$$\begin{aligned} \Vert \widehat{{\dot{S}}_h}(z)P_h\Vert _{\mathcal {L}(H_0)}\le M \end{aligned}$$

for all $z\in \Sigma _\omega$ with $\omega <\pi /(1+\alpha )$. Hence, with $\Gamma$ as above, we have

$$\begin{aligned} \Vert {\dot{S}}_h(t)P_h\Vert _{\mathcal {L}(H_0)}=\left\| \frac{1}{2\pi i}\int _\Gamma e^{tz}\widehat{{\dot{S}}_h}(z)P_h\,dz\right\| _{\mathcal {L}(H_0)}\le M\int _\Gamma e^{-ct|z|}\, |dz|\le {\tilde{M}} t^{-1},\,t>0, \end{aligned}$$

and the proof is complete. $\square$

We can now prove an error estimate in Hölder norms.

#### Theorem 3.11

Let *U* and $U_h$ be given by (31) and (34), respectively. Set ${e}(t) := U(t)-U_h(t)$ and $e_0(t):=S(t)U_0-S_h(t)P_hU_0$.

- Let $p>2$ and $0< \gamma _1<\frac{1}{2}-\frac{1}{p}$. Then, for $\gamma <\frac{1}{2}-\frac{1}{p}-\gamma _1$ and $\beta <\frac{\gamma _1}{\alpha +1}$ the error estimate
  43 $$\begin{aligned}&\Vert e\Vert _{L^p(\Omega ;C^\gamma ([0,T];H_0))}\le C(T,p,\beta ,\gamma )\nonumber \\&\quad \left( \Vert e_0\Vert _{L^p(\Omega ;C^\gamma ([0,T];H_0))} +\Vert e(0)\Vert _{L^p(\Omega ;H_0)}+Ch^{2\beta }\right) \end{aligned}$$
  holds. If the mesh is quasi-uniform and $U_0\in L^p(\Omega ;D(A^{\frac{1}{1+\alpha }}))$, then
  44 $$\begin{aligned} \Vert e\Vert _{L^p(\Omega ;C^\gamma ([0,T];H_0))}\le C(T,p,\beta ,\gamma ,U_0)h^{2\beta }. \end{aligned}$$
- Let $p>2$. Then, for $\beta <\frac{\frac{1}{2}-\frac{1}{p}}{\alpha +1}$ the error estimate
  45 $$\begin{aligned} \Vert e\Vert _{L^p(\Omega ;C([0,T];H_0))}\le C(T,p,\beta ,\gamma )\left( \Vert e_0\Vert _{L^p(\Omega ;C([0,T];H_0))} +Ch^{2\beta }\right) \end{aligned}$$
  holds. If $U_0\in L^p(\Omega ;D(A^{\beta (1+\epsilon )}))$ for some $\epsilon >0$, then
  46 $$\begin{aligned} \Vert e\Vert _{L^p(\Omega ;C([0,T];H_0))}\le C(T,p,\beta ,\gamma ,U_0)h^{2\beta }. \end{aligned}$$

#### Proof

Let $$0< \gamma _1<\frac{1}{2}-\frac{1}{p}$$. Then,

47 $$\begin{aligned} t^{\gamma _1}\Vert {\dot{S}}(t)x\Vert _{H_0}+t^{\gamma _1-1}\Vert S(t)x\Vert _{H_0}\le Ct^{\gamma _1-1}\Vert x\Vert _{H_0}:=s_1(t)\Vert x\Vert _{H_0} \end{aligned}$$

and thus $s_1\in L ^1 ((0,T];\mathbb {R}^+_0)$. Furthermore, by Proposition 3.10,

48 $$\begin{aligned} t^{\gamma _1}\Vert {\dot{E}}_h(t)x\Vert _{H_0}+t^{\gamma _1-1}\Vert E_h(t)x\Vert _{H_0}\le Ch^{2\beta }t^{-{\beta }(\alpha +1)-1+\gamma _1}\Vert x\Vert _{H_0}:=h_1(t)h^{2\beta }\Vert x\Vert _{H_0},\nonumber \\ \end{aligned}$$

for $0<h<1$. We have that $h_1\in L ^1 ((0,T];\mathbb {R}^+_0)$ if and only if $-{\beta }(\alpha +1)-1+\gamma _1>-1$; that is, when $\beta <\frac{\gamma _1}{\alpha +1}$. Then, the error bound in (43) follows from Theorem 2.11 for each fixed $0<h<1$ with $\Psi _{n}^1=h^{-2\beta }E_{h}$, $r_1(n)=h^{2\beta }$ for all $n\in \mathbb {N}$ and $\Psi _n^2=0$, $r_2(n)=h^{2\beta }$ for all $n\in {\mathbb {N}}$ using (47) and (48) and noting that the function $h_1$ in (48) is independent of *h* and then so is the constant *C* in the error estimate (13) of Theorem 2.11. To show (44) note that, by a standard finite element estimate, we have

49 $$\begin{aligned} \Vert e(0)\Vert _{L^p(\Omega ;H_0)}=\Vert (I-P_h)U_0\Vert _{L^p(\Omega ;H_0)}\le Ch^{\frac{2}{1+\alpha }}\Vert A^{\frac{1}{1+\alpha }}U_0\Vert _{L^p(\Omega ;H_0)}. \end{aligned}$$

Furthermore, for $x\in H_0$, by choosing $\chi =P_hx$ in (35), we see that the function $t\mapsto S_h(t)P_hx$ is the unique solution of (35); that is,

$$\begin{aligned} {\dot{S}}_h(t)P_hx+\frac{1}{\Gamma (\alpha )}\int _0^t (t-s)^{\alpha -1}A_hS_h(t)P_hx\,d s=0,\,t>0, \end{aligned}$$

and therefore it follows that

$$\begin{aligned} \Vert \dot{S_h}(t)P_hx\Vert _{H_0}\le Ct^\alpha \Vert A_hP_hx\Vert _{H_0}, \end{aligned}$$

where we used the stability estimate $\Vert S_h(t)P_h\Vert _{\mathcal {L}(H_0)}\le C$, $t\ge 0$. Using also (41) we conclude, by interpolation that

50 $$\begin{aligned} \Vert {\dot{S}}_h(t)P_hx\Vert _{H_0}\le Ct^{\mu (1+\alpha )-1}\Vert A_h^{\mu }P_hx\Vert _{H_0}, \,\mu \in [0,1]. \end{aligned}$$

Therefore, using (33) and (50) with $\mu =\frac{1}{1+\alpha }$, it follows that

$$\begin{aligned}&\Vert {\dot{E}}_h(t)x\Vert _{H_0}\le \Vert {\dot{S}}_h(t)P_hx\Vert _{H_0}+ \Vert {\dot{S}}(t)x\Vert _{H_0}\le {C}\Vert A_h^{\frac{1}{1+\alpha }}P_hx\Vert _{H_0}+{C}\Vert A^{\frac{1}{1+\alpha }}x\Vert _{H_0}\\&\quad = {C}\Vert A_h^{\frac{1}{1+\alpha }}P_hA^{-\frac{1}{1+\alpha }}A^{\frac{1}{1+\alpha }}x\Vert _{H_0}+{C}\Vert A^{\frac{1}{1+\alpha }}x\Vert _{H_0} \\&\quad \le C\Vert A_h^{\frac{1}{1+\alpha }}P_hA^{-\frac{1}{1+\alpha }}\Vert _{\mathcal {L}(H_0)}\Vert A^{\frac{1}{1+\alpha }}x\Vert _{H_0} +{C}\Vert A^{\frac{1}{1+\alpha }}x\Vert _{H_0}\le C \Vert A^{\frac{1}{1+\alpha }}x\Vert _{H_0}, \,t\in [0,T]. \end{aligned}$$

Here we also used that, by the self-adjointness of $A^{-1},A_h$ and $P_h$,

$$\begin{aligned} \Vert A_h^{\delta }P_hA^{-\delta }\Vert _{\mathcal {L}(H_0)}&=\Vert P_hA_h^{\delta }P_hA^{-\delta }\Vert _{\mathcal {L}(H_0)}=\Vert (P_hA_h^{\delta }P_hA^{-\delta })^{*}\Vert _{\mathcal {L}(H_0)}\\&=\Vert (A^{-\delta })^*(P_hA_h^{\delta }P_h)^*\Vert _{\mathcal {L}(H_0)}=\Vert A^{-\delta }A_h^{\delta }P_h\Vert _{\mathcal {L}(H_0)}\le C, \end{aligned}$$

where the last inequality holds for $\delta \in [0,1]$ for quasi-uniform meshes, see, for example, the proof of Theorem 4.4 (iv) in [36]. Thus, using interpolation in Hölder spaces and the smooth data estimate from (36),

$$\begin{aligned} \Vert e_0\Vert _{C^\gamma ([0,T];H_0)}\le C \Vert e_0\Vert _{C^1([0,T];H_0)}^{\gamma }\Vert e_0\Vert _{C([0,T];H_0)}^{1-\gamma }\le C h^{(1-\gamma )\frac{1}{1+\alpha }\frac{2}{1+\epsilon }}\Vert A^{\frac{1}{1+\alpha }}U_0\Vert _{H_0}. \end{aligned}$$

As

$$\begin{aligned} (1-\gamma )\frac{1}{1+\alpha }\frac{2}{1+\epsilon }>\frac{2\gamma _1}{1+\epsilon }\frac{1}{1+\alpha }+\left( \frac{1}{p}+\frac{1}{2}\right) \frac{1}{1+\alpha }\frac{2}{1+\epsilon }>\frac{2\gamma _1}{1+\epsilon }\frac{1}{1+\alpha } \end{aligned}$$

it follows that

$$\begin{aligned} \Vert e_0\Vert _{L^p(\Omega ;C^\gamma ([0,T];H_0))}\le Ch^{2\beta } \Vert A^{\frac{1}{1+\alpha }}U_0\Vert _{L^p(\Omega ;H_0)} \end{aligned}$$

for all $\beta <\frac{\gamma _1}{\alpha +1}$ and the proof of (44) is complete in view of (43) and (49). Next, the estimate (45) follows from Corollary 2.12 in view of (47) and (48). Finally, using (36), we immediately conclude that

$$\begin{aligned} \Vert e_0\Vert _{L^p(\Omega ;C([0,T];H_0))}\le Ch^{2\beta } \Vert A^{\beta (1+\epsilon )}U_0\Vert _{L^p(\Omega ;H_0)}, \end{aligned}$$

which finishes the proof of (46) in view of (45) and the proof of the theorem is complete. $\square$

#### Remark 3.12

If $U_0$ is deterministic, then we may take *p* arbitrarily large in Theorem 3.11 (similarly, in Theorem 3.4 in case $u_0$ and $u_1$ are deterministic). We also point out that the estimate on $\Vert e_0\Vert _{C^\gamma ([0,T];H_0)}$ in the proof of Theorem 3.11 is not sharp in terms of the regularity of the initial data. This follows from the fact that we estimate the $\gamma$-Hölder norm by interpolation and not directly and hence more regularity on $U_0$ is assumed than what is necessary. However, a sharp, direct estimate on $\Vert e_0\Vert _{C^\gamma ([0,T];H_0)}$ is not available in the finite element literature, and a derivation would be beyond the scope of this paper.

#### Remark 3.13

(Stochastic heat equation) Here we briefly comment on the stochastic heat equation which also fits in our abstract framework. Suppose that *F* and *G* are as above and $S(t):=e^{-tA}$ is the heat semigroup and $S_h(t):=e^{-tA_h}P_h$, $t\ge 0$. In this case the well-known error estimates, see [55, Chapter 3],

$$\begin{aligned}&\Vert E_h(t)x\Vert _{H_0}\le Ch^\beta \Vert A^{\frac{\beta }{2}}x\Vert _{H_0},\quad \beta \in [0,2],\, x\in \mathcal {D}(A^{\beta }),\,t\in [0,T];\\&\Vert E_h(t)\Vert _{\mathcal {L}(H_0)}\le Ch^{2\beta }t^{-\beta },\quad \beta \in [0,1],\,t\in (0,T];\\&\Vert {\dot{E}}_h(t)\Vert _{\mathcal {L}(H_0)} \le Ch^{2\beta }t^{-\beta -1},\quad \beta \in [0,1],\,t\in (0,T], \end{aligned}$$

hold for $0<h< 1$. Note that these are essentially (36–38) for $\alpha =0$. Then, similarly as in the proof of Theorem 3.11 we get, for $p>2$, $0< \gamma _1<\frac{1}{2}-\frac{1}{p}$, $\gamma <\frac{1}{2}-\frac{1}{p}-\gamma _1$ and $\beta <\gamma _1$ that the error estimate

$$\begin{aligned} \Vert e\Vert _{L^p(\Omega ;C^\gamma ([0,T];H_0))}\le C(T,p,\beta )\left( \Vert e_0\Vert _{L^p(\Omega ;C^\gamma ([0,T];H_0))} +\Vert e(0)\Vert _{L^p(\Omega ;H_0)}+Ch^{2\beta }\right) \end{aligned}$$

holds, where ${e}(t) := U(t)-U_h(t)$. In particular we have

$$\begin{aligned} \Vert e\Vert _{L^p(\Omega ;C([0,T];H_0))}\le C(T,p,\beta )\left( \Vert U_0\Vert _{L^p(\Omega ;A^{\beta })} +1\right) h^{2\beta } \end{aligned}$$

for $\beta <\frac{1}{2}-\frac{1}{p}$. This result is consistent with [16, Proposition 4.2] but less smoothness on the noise is assumed here; that is, we may take $\delta _G=0$.

#### Remark 3.14

In [40], a simplified version of (30) was considered with $\Gamma (u)=I$ and $F=0$ (linear equation, additive noise). It was shown there that if *Q* has finite trace then

$$\begin{aligned} \sup _{t\in [0,T]}\Vert e(t)\Vert _{L^2(\Omega ;H_0)}\le C\left( \Vert U_0\Vert _{L^2(\Omega ;A^{\frac{1+\epsilon }{2(1+\alpha )}})} +1\right) h^{\frac{1}{1+\alpha }}. \end{aligned}$$

This is consistent with Theorem 3.11 as in this case we may first take $U_0=0$ and hence take *p* in (45) arbitrarily large and then add the estimate for the initial term.

## Numerical experiments

In this section, we will illustrate our theoretical results by some numerical experiments. The underlying equation we consider is the fractional stochastic wave Eq. (30), where $\mathcal {D}=[0,1]$, $F=0$, $\Gamma (U)=I$, and $A=-\Delta$ is the Laplacian with Dirichlet boundary conditions in $H_0=(L^2(\mathcal {D}),\Vert \cdot \Vert )$ with inner product denoted by $(\cdot ,\cdot )_{}$. In particular, we will implement the numerical solution for the following equation:

51 $$\begin{aligned} \left\{ \begin{aligned}&dU(t,x)-\int _0^tb(t-s)\Delta U(s,x)\,ds\,dt= Q^{\frac{1}{2}}d {W_H}(t,x),\,t\in (0,1],\,x\in \mathcal {D}; \\&U(0,x)=\sin (\pi x):=U_0(x),\,x\in \mathcal {D}, \end{aligned} \right. \end{aligned}$$

where $W_H$ is a *H*-cylindrical Wiener process with $H=H_0$, $b(t)=t^{\alpha -1}/\Gamma (\alpha ),$ $\alpha \in (0,1)$, and $Q:H\rightarrow H$ is symmetric, bounded, and positive semidefinite.

In Subsection 4.1, we apply the spectral Galerkin method based on the eigenvalues $\lambda _k=k^2\pi ^2$ and the orthonormal basis of corresponding eigenfunctions $\{e_k:k\in \mathbb {N}\}$. For the driving noise we take space time white noise; that is, $Q=I$. In particular, we take $W_H$ to be given by the formal series $W_{{H}}(t,x)=\sum _{k=1}^{\infty } e_k(x)\beta ^k(t)$, $x\in \mathcal {D}$, $t\ge 0$, where $\{\beta ^k:k=1,2,\dots \}$ is a family of mutually independent standard scalar Brownian motions. To perform the integration in time, we use the Mittag-Leffler Euler integrator (MLEI) method, developed for semilinear problems in [38]. In the present linear setting this method is exact, that is, no additional time-discretization error is introduced and we may simulate the spatially approximated process exactly on a time-grid.

In Subsection 4.2, we approximate the solution of (51) by finite elements. We consider a Wiener process which is of trace class given by

52 $$\begin{aligned} Q^{\frac{1}{2}}{W_H(t,x)}:=1_{[0,0.5]}(x)\beta (t), {x\in \mathcal {D}, t\in [0,1],} \end{aligned}$$

where $\beta$ is a scalar Brownian motion and $1_{[0,0.5]}$ is the characteristic function of the interval [0, 0.5]. That is, the Fourier expansion of the driving Wiener process contains a single term only and thus its covariance operator is of rank 1 and hence trace class. The motivation for the particular choice of the Wiener process is to consider trace class noise which does not possess additional spatial smoothness. This is needed so that we do not observe higher convergence rate, due to additional regularity, in the numerical experiments than predicted by the theory for trace class noise. To perform the time integration we implement a Lubich Convolution Quadrature (LCQ) method, for details see [44, 45]. This method was successfully applied to a similar problem of the third author in [40]. The LCQ method is easier to implement than the MLEI in case of finite elements and a correlated noise.

### The spectral Galerkin method and the MLEI-method

The mild solution of (51) with space time white noise can be written as

53 $$\begin{aligned} U(t)=S(t)U_0+\sum _{k=1}^{\infty } \int _0^tS(t-\tau ) e_k ~d\beta ^k(\tau ), \end{aligned}$$

where, as shown in [38], the resolvent family $\{S(t)\}_{t\ge 0}$ can be represented as

54 $$\begin{aligned} S(t)v=\sum \limits _{k=1}^{\infty } E_{\alpha +1}(-\lambda _kt^{\alpha +1})(v,e_k)e_k,\, t>0, \end{aligned}$$

where $E_{\rho }(z)$, $\rho >0$, is the one parameter Mittag-Leffler function (MLF) defined by

$$\begin{aligned} E_{\rho }(z):=\sum \limits _{k=0}^{\infty } \frac{z^k}{\Gamma (\rho k+1)},\quad z\in \mathbb {C}. \end{aligned}$$

For more details about Mittag-Leffler function and their application, we refer to the paper [49] and the book [25]. Moreover, in order to implement the MLF, we use the Matlab function mlf.m, see [50].

Let $\Pi =\{0=t_0<t_1<\cdots <t_M=1\}$ be a partition of the time interval [0, 1]. From the representation given in (53) we get for $m=0,1,2,3,\dots ,M$

$$\begin{aligned} U(t_m)=S(t_m)U_0+\sum _{k=1}^{\infty } \int _0^{t_m}S(t_m-s) e_k ~d\beta ^k(s). \end{aligned}$$

For the discretization in space, we introduce the finite dimensional subspaces ${H^N}=\text {span}\{e_k:k=1,2,\dots ,N \}$ of *H* and the orthogonal projection $\mathcal {P}_N:H\rightarrow {H^N}$ given by

$$\begin{aligned} \mathcal {P}_N v= \sum \limits _{k=1}^{N}(v,e_k)e_k,\quad v\in H. \end{aligned}$$

Using (54) we then get

$$\begin{aligned} S_N(t) v:= S(t)\mathcal {P}_N v =\sum \limits _{k=1}^{N} E_{\alpha +1}(-\lambda _kt^{\alpha +1})(v,e_k)e_k. \end{aligned}$$

This way we obtain for the approximation ${{\bar{U}}}^N_m$ of $U(t_m)$ given by (53) by the Galerkin method

55 $$\begin{aligned} {{\bar{U}}}_m^N=S_N(t_m){{\bar{U}}}_0^N+\sum _{k=1}^{N} \int _0^{t_m}S_N(t_m-s) e_k ~d\beta ^k(s), \end{aligned}$$

with initial value ${{\bar{U}}}_0^N=\mathcal {P}_N U_0$. Let us define ${{\bar{U}}}_{m,k}^N$ by

$$\begin{aligned} {{\bar{U}}}_{m,k}^N=E_{\alpha +1}(-\lambda _kt_m^{\alpha +1}) \bar{U}^N_{0,k}+ \mathcal {O}_k(t_m), \end{aligned}$$

where $${{\bar{U}}}^N_{0,k}=(U(0),e_k)$$ and

$$\begin{aligned} \mathcal {O}_k(t_m):=\int _0^{t_m}E_{\alpha +1}(-\lambda _k(t_m-s)^{\alpha +1}) ~d\beta ^k(s). \end{aligned}$$

Then, (55) can be rewritten as

$$\begin{aligned} {{\bar{U}}}_m^N=\sum \limits _{k=1}^{N} {{\bar{U}}}_{m,k}^{N}e_k. \end{aligned}$$

To simulate the stochastic convolution process let us observe that

$$\begin{aligned} \mathcal {N}:= (\mathcal {O}_k(t_1),\mathcal {O}_k(t_2),\dots ,\mathcal {O}_k(t_M))^\top \end{aligned}$$

is a *M*-dimensional Gaussian random variable with zero mean and covariance matrix $$ R=(R_{i,j} )_{i,j=1}^{M}$$

$$\begin{aligned} R_{i,j}=\int _0^{t_i\wedge t_j}E_{\alpha +1}(-\lambda _k(t_i-s)^{\alpha +1})E_{\alpha +1}(-\lambda _k(t_j-s)^{\alpha +1}) ~ds. \end{aligned}$$

Thus, $\mathcal {N}$ can be represented as $K\chi$, where $\chi$ is an *M*-dimensional standard Gaussian random variable and *K* is the solution of equation $KK^T=R$ (see Theorem 2.2 of [28]); the equation $KK^T=R$ can be solved by the Cholesky factorization.

**Fig. 1:**
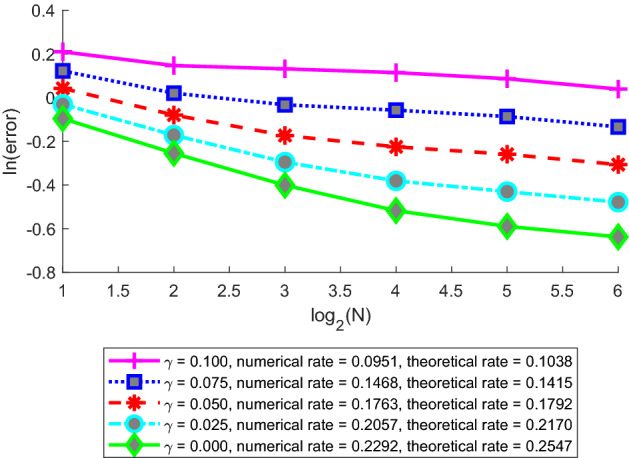
The approximation error for the spectral Galerkin method and the MLEI-method in the $L^2(\Omega ;C^\gamma ([0,T];H_0))$-norm with $\alpha =0.325$

**Fig. 2:**
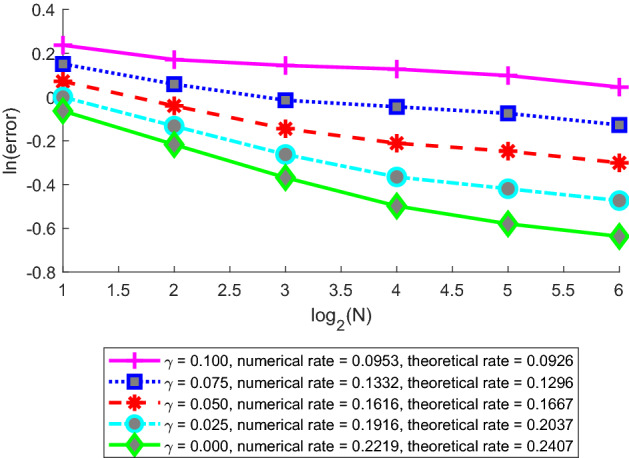
The approximation error for the spectral Galerkin method and the MLEI-method in the $L^2(\Omega ;C^\gamma ([0,T];H_0))$-norm with $\alpha =0.35$

**Fig. 3:**
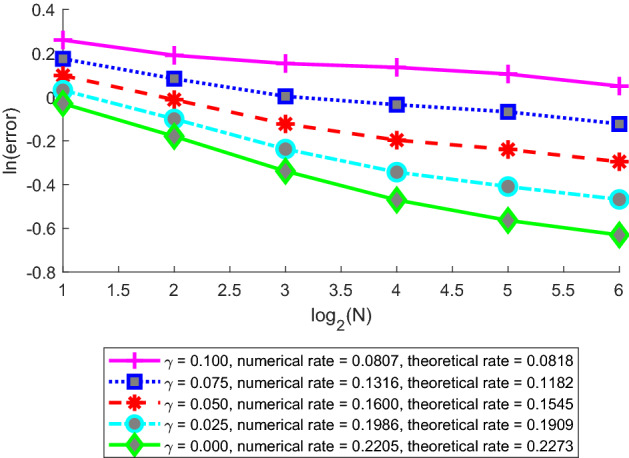
The approximation error for the spectral Galerkin method and the MLEI-method in the $L^2(\Omega ;C^\gamma ([0,T];H_0))$-norm with $\alpha =0.375$

In our numerical experiment, we simulated 100 sample paths to verify the rate of convergence in the $L^2(\Omega ;C^\gamma ([0,T];H_0))$-norm for different $\gamma$ and $\alpha$. According to Example 3.8 we expect theoretical rate of $\nu <\frac{\gamma _1}{1+\alpha }-\delta _G$ in the $L^p(\Omega ;C^\gamma ([0,T];H_0))$-norm for appropriately smooth and integrable initial data $U_0$, where $\gamma <\frac{1}{2}-\frac{1}{p}-\gamma _1$ and $p>2$. Note that the parameter $\alpha$ in Example 3.8 corresponds to $\alpha +1$ in the present example, see Remark 3.9. Taking into account that $\lambda _N=N^2\pi ^2$ and that $Q=I$ and hence $\delta _G>\frac{1}{4}$ we obtain a rate in *N* of almost $2(\tfrac{1}{2}-\tfrac{1}{p}-\gamma )/(1+\alpha )-\frac{1}{2}$. Note that since $U_0$ is a deterministic eigenfunction of *A* and thus $U_0\in L^p(\Omega ;\mathcal {D}(A^s))$ for any $p>2$ and $s\ge 0$, we may bound the $L^2(\Omega ;C^\gamma ([0,T];H_0))$-norm by the $L^p(\Omega ;C^\gamma ([0,T];H_0))$-norm for any $p>2$ and hence we expect a rate in *N* of almost $(1-2\gamma )(1+\alpha )-\frac{1}{2}$ in the $L^2(\Omega ;C^\gamma ([0,T];H_0))$-norm. In the simulations, we chose a small time step $\Delta t=t_k-t_{k-1}=0.001$, $k=0,1,\dots ,M$ and vary the dimension of the finite dimensional approximation space ${H^{N_i}}$, $i=1,2,\dots ,6$, with $N_i=2^i$. To estimate the error, we computed a reference solution with $N=2^{13}$. In Fig. 1, we present the error of the numerical approximation in the $L^2(\Omega ;C^\gamma ([0,T];H_0))$-norm for $\alpha =0.325$ with varying $\gamma$ (see also Fig. 2 and Fig. 3 for $\alpha =0.35$ and $\alpha =0.375$, respectively). In Figs. 1-3, we also compute the numerical rate of convergence given by

56 $$\begin{aligned} \min _{i=1,2,3,4,5}-\frac{\ln \left( \frac{\text {error}_{\gamma }(N_i)}{\text {error}_{\gamma }(N_{i+1})} \right) }{\ln \left( \frac{N_i}{N_{i+1}}\right) }, \end{aligned}$$

for $\gamma =0,0.025,0.05,0.075,0.1$ where $\text {error}_{\gamma }(N_i)$ is the error of the numerical approximation in the $L^2(\Omega ;C^\gamma ([0,T];H_0))$-norm when the dimension of $H^{N_i}$ is $N_i$. Here, one may observe that if $\gamma$ decreases, then the rate of convergence increases. Moreover, Figures 1-3 also show that the numerical rate of convergence is close to the theoretical rate.

### The finite element method and the LCQ-method

We first perform a time discretization of (51) with $Q^{\frac{1}{2}}W_H$ given by (52) by the first order LCQ-method, for more details see e.g. [40]. To describe the first order LCQ method, let $\Pi =\{0=t_0<t_1<t_2,\cdots <t_M=1\}$ be an equidistant partition of the time interval [0, 1] with time step size $\Delta t=t_{m}-t_{m-1}$, $m=1,2,3,\dots , M$. The approximation of a convolution term

$$\begin{aligned} \int _0^{t_m} b(t_m-s)g(s)\, ds \end{aligned}$$

is then given by

$$\begin{aligned} \sum _{i=1}^m\omega _{m-i}g(t_i), \end{aligned}$$

where the weights $\{\omega _k:k\in \mathbb {N}\cup \{0\}\}$ are chosen such that

$$\begin{aligned} \sum _{k=0}^\infty \omega _kz^k={{\widehat{b}}}\left( \frac{1-z}{\Delta t}\right) ,\quad |z|<1. \end{aligned}$$

This is a first order quadrature; that is, it has an approximation order of $\mathcal {O}(\Delta t)$. Applying the LCQ-method, the equation for the approximation ${{\bar{U}}}$, where ${{\bar{U}}}_n(x)\approx U(t_n,x)$, can be written as follows

57 $$\begin{aligned} {{\bar{U}}}_n-{{\bar{U}}}_{n-1}+\Delta t\Big (\sum \limits _{i=1}^{n}\omega _{n-i}A {{\bar{U}}}_i \Big )=1_{[0,0.5]} \Delta _n \beta ,\quad {{\bar{U}}}_0=U(0), \end{aligned}$$

where $$\Delta _n \beta =\beta (t_n)-\beta (t_{n-1})$$, $$n=1,2,\dots , M$$.

Secondly, we discretize (57) by linear finite elements. Let us consider a partition of the domain $\mathcal {D}=[0,1]$ given by $\{0=x_0<x_1<x_2<\cdots <x_N=1\}$ with constant mesh size $h=x_{m+1}-x_{m}$, $m=0,1,\dots , N-1$. Let us denote the finite element spaces by $\{ V_h \}_{0<h<1}$, where $V_h=\text {span}\{\varphi _k:k=1,2,\dots ,N-1 \}\subset {H^1_0(\mathcal {D})}$ with $\varphi _k$ being a standard hat function in the 1-D finite element method [42]. We introduce the discrete Laplacian

58 $$\begin{aligned} A_{h}:V_h\rightarrow V_h, \quad (A_{h} \xi , \chi )_{} = ( \xi ',\chi ')_{},\quad \xi ,\chi \in V_h, \end{aligned}$$

where $v'=\frac{dv}{dx}$ denotes the derivative, and the orthogonal projection

$$\begin{aligned} P_{h}: H_0 \rightarrow V_h,\quad (P_{h} f, \chi )_{} = ( f,\chi )_{},\quad f\in H_0,~ \chi \in V_h. \end{aligned}$$

In order to obtain the numerical formulation for (51), we compute a $V_h$-valued random variable ${{\bar{U}}}^h_{n}$ satisfying for all $k=1,2,\dots ,N-1$

$$\begin{aligned} \left\{ \begin{aligned} ({{\bar{U}}}_n^h,\varphi _k)&= ({{\bar{U}}}_{n-1}^h,\varphi _k)- \Delta t\Big (\sum \limits _{i=1}^{n}\omega _{n-i}(A_h\bar{U}^h_{i},\varphi _k)\Big )+ (1_{[0,0.5]} ,\varphi _k)\Delta _n \beta ; \\ ({{\bar{U}}}^h_0,\varphi _k)&=(\sin (\pi x),\varphi _k), \end{aligned} \right. \end{aligned}$$

where ${{\bar{U}}}_n^h(x)=\sum \limits _{k=1}^{N-1} \bar{U}_{n,k}^h\varphi _k(x)\approx U(t_n,x)$. From (58) we then obtain

$$\begin{aligned} \left\{ \begin{aligned}&{\sum \limits _{m=1}^{N-1} [(\varphi _m,\varphi _k)+\Delta t \omega _0 (\varphi '_m,\varphi '_k)] \bar{U}_{n,m}^h}\\&= \sum \limits _{m=1}^{N-1} (\varphi _m,\varphi _k){{\bar{U}}}_{n-1,m}^h -\Delta t \sum \limits _{m=1}^{N-1} ( \varphi '_m,\varphi '_k) \Big (\sum \limits _{i=1}^{n-1}\omega _{n-i} {{\bar{U}}}_{i,m}^h \Big )+ (1_{[0,0.5]} ,\varphi _k)\Delta _n \beta ; \\&\sum \limits _{m=1}^{N-1} (\varphi _m,\varphi _k) {{\bar{U}}}_{0,m}^h =(\sin (\pi x),\varphi _k). \end{aligned} \right. \end{aligned}$$

The above system can be rewritten in the following form

$$\begin{aligned} \varvec{{{\bar{U}}}_n^h}= ({\varvec{K}}+\Delta t \omega _0 {\varvec{L}} )^{-1}({\varvec{K}} \varvec{\bar{U}_{n-1}^h}-\Delta t \sum _{i=1}^{n-1}\omega _{n-i}{\varvec{L}} \varvec{{{\bar{U}}}_i^h}+{\varvec{J}}\Delta _n\beta ). \end{aligned}$$

Here, the vectors $\varvec{{{\bar{U}}}_n^h}$ and ${\varvec{J}}$ are defined by $\varvec{{{\bar{U}}}_n^h}=({{\bar{U}}}_{n,1}^h,\dots ,\bar{U}_{n,N-1}^h)^\top$ and ${\varvec{J}}=(J_1, J_2,\dots , J_{N-1})^\top$ where $J_k=(1_{[0,0.5]} ,\varphi _k)$ for $k=1,\dots ,N-1$. Moreover, the stiffness matrix $\varvec{K}=(K_{i,j} )_{i,j=1}^{N-1}$ and the mass matrix $\varvec{L}=(L_{i,j} )_{i,j=1}^{N-1}$ are given by

$$\begin{aligned} K_{i,j}=\int \limits _{0}^{1}\varphi _i(x)\varphi _j(x)~dx,\quad L_{i,j}=\int \limits _{0}^{1}\varphi '_i(x)\varphi '_j(x)~dx, \end{aligned}$$

respectively.

**Fig. 4:**
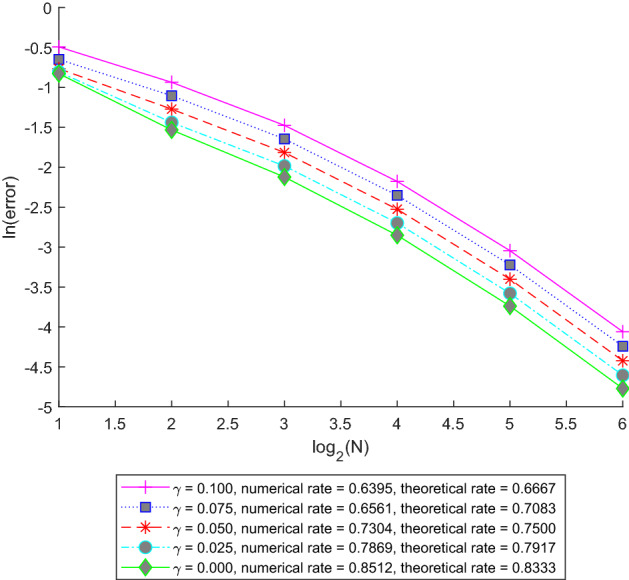
The approximation error for the finite element method and the LCQ-method in the $L^2(\Omega ;C^\gamma ([0,T];H_0))$-norm with $\alpha =0.2$

**Fig. 5:**
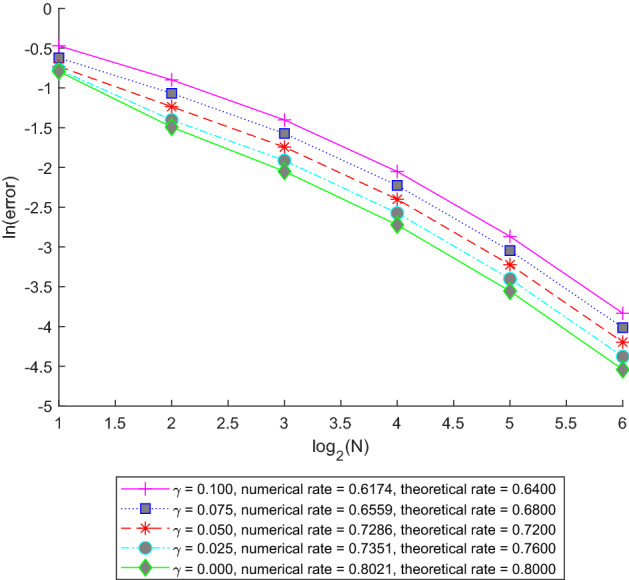
The approximation error for the finite element method and the LCQ-method in the $L^2(\Omega ;C^\gamma ([0,T];H_0))$-norm with $\alpha =0.25$

**Fig. 6:**
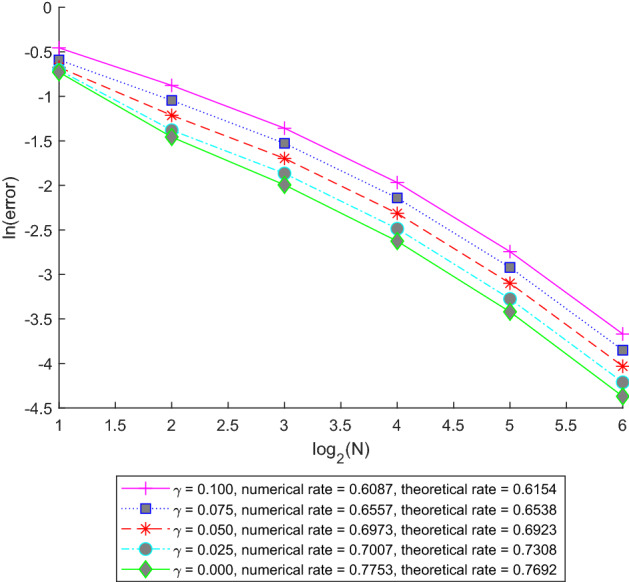
The approximation error for the finite element method and the LCQ-method in the $L^2(\Omega ;C^\gamma ([0,T];H_0))$-norm with $\alpha =0.3$

In our numerical experiment, we used 500 sample paths to verify the dependence of the rate of convergence in the $L^2(\Omega ;C^\gamma ([0,T];H_0))$-norm on $\gamma$ and $\alpha$. The theoretical rate of convergence is almost $(1-2\gamma )/(1+\alpha )$ according to Theorem 3.11. Note again that, similarly to the previous example, since $U_0$ is a deterministic eigenfunction of *A*, we may bound the $L^2(\Omega ;C^\gamma ([0,T];H_0))$-norm by the $L^p(\Omega ;C^\gamma ([0,T];H_0))$-norm for any $p>2$. In the simulations we choose fixed a step time $\Delta t=0.0005$ and varying the dimension of the space approximation $\dim V_h=N_i-1=2^i$, $i=1,2,\dots ,6$; that is, we take $h=\frac{1}{N_i}$, $i=1,2,\dots , 6$. Then, in order to measure the error, we computed a reference solution with a mesh size $h=\frac{1}{2^{11}}$. In Fig. 4, we present the error of the numerical approximation in the $L^2(\Omega ;C^\gamma ([0,T];H_0))$-norm for $\alpha =0.2$ with varying values of $\gamma$ (see also Fig. 5 and Fig. 6 for $\alpha =0.25$ and $\alpha =0.3$, respectively). Similarly to Sect. 4.1, in this section we also compute the numerical rate of convergence according to (56) for $\gamma =0,0.025,0.05,0.075,0.1$. Here, one may observe that if $\gamma$ decreases, then the rate of convergence again increases. Moreover, the figures also show that the numerical rate of convergence is close to the theoretical rate.

## References

[1] Andersson A, Kovács M, Larsson S (2016). Weak error analysis for semilinear stochastic Volterra equations with additive noise. J. Math. Anal. Appl..

[2] Arendt, W., Batty, C.J.K., Hieber, M., Neubrander, F.: Vector-valued Laplace Transforms and Cauchy Problems. In: Monographs in Mathematics 96. Birkhäuser (2001)

[3] Baeumer B, Geissert M, Kovács M (2015). Existence, uniqueness and regularity for a class of semilinear stochastic Volterra equations with multiplicative noise. J. Diff. Equ..

[4] Bally V, Millet A, Sanz-Solé M (1995). Approximation and support theorem in Hölder norm for parabolic stochastic partial differential equations. Ann. Probab..

[5] Barbu V, Bonaccorsi S, Tubaro L (2014). Existence and asymptotic behavior for hereditary stochastic evolution equations. Appl. Math. Optim..

[6] Bonaccorsi S (2009). Fractional stochastic evolution equations with Lévy noise. Diff. Int. Equ..

[7] Bonaccorsi S, Da Prato G, Tubaro L (2012). Asymptotic behavior of a class of nonlinear stochastic heat equations with memory effects. SIAM J. Math. Anal..

[8] Bonaccorsi S, Desch G (2013). Volterra equations in Banach spaces with completely monotone kernels. NoDEA Nonlinear. Diff. Equ. Appl..

[9] Bonaccorsi S, Fantozzi M (2006). Infinite dimensional stochastic Volterra equations with dissipative nonlinearity. Dynam. Syst. Appl..

[10] Bonaccorsi S, Mastrogiacomo E (2009). An analytic approach to stochastic Volterra equations with completely monotone kernels. J. Evol. Equ..

[11] Bonaccorsi S, Tubaro L (2003). Mittag-Leffler’s function and stochastic linear Volterra equations of convolution type. Stochastic Anal. Appl..

[12] Da Prato G, Kwapien S, Zabczyk J (1987). Regularity of solutions of linear stochastic equations in Hilbert spaces. Stochastics.

[13] Clément P, Da Prato G, Prüss J (1997). White noise perturbation of the equations of linear parabolic viscoelasticity. Rend. Istit. Mat. Univ. Trieste.

[14] Clément P, Desch W, Homan KW (2002). An analytic semigroup setting for a class of Volterra equations. J. Integral Equ. Appl..

[15] Clément P, Londen SO, Simonett G (2004). Quasilinear evolutionary equations and continuous interpolation spaces. J. Diff. Equ..

[16] Cox S, Hausenblas E (2012). Pathwise space approximations of semi-linear parabolic SPDEs with multiplicative noise. Int. J. Comput. Math..

[17] Cox S, Hausenblas E (2013). A perturbation result for semi-linear stochastic differential equations in UMD Banach spaces. J. Evol. Equ..

[18] Cox S, Hutzenthaler M, Jentzen A, van Neerven J, Welti T (2021). Convergence in Hölder norms with applications to Monte Carlo methods in infinite dimensions. IMA J. Num. Anal..

[19] Cox S, van Neerven J (2013). Pathwise Hölder convergence of the implicit-linear Euler scheme for semi-linear SPDEs with multiplicative noise. Numer. Math..

[20] Dareiotis KA, Gyöngy I (2014). A comparison principle for stochastic integro-differential equations. Potential Anal..

[21] Desch, W., Londen, S. O.: On a stochastic parabolic integral equation. In: Functional analysis and evolution equations, pp. 157–169. Birkhäuser, Basel (2008)

[22] Desch W, Londen SO (2011). An $$L_p$$-theory for stochastic integral equations. J. Evol. Equ..

[23] Desch, W., Londen, S. O.: Semilinear stochastic integral equations in $$L_p$$. In: Parabolic problems, volume 80 of Progr. Nonlinear Differential Equations Appl., pp. 131–166. Birkhäuser/Springer Basel AG, Basel (2011)

[24] Foondun M, Nane E (2017). Asymptotic properties of some space-time fractional stochastic equations. Math. Z..

[25] Gorenflo, R., Kilbas, A.A., Mainardi, F., Rogosin, S.V.: Mittag-Leffler Functions, Related Topics and Applications. In Springer Monographs in Mathematics, Springer-Verlag, Berlin, Heidelberg. (2014)

[26] Gunzburger M, Li B, Wang J (2019). Convergence of finite element solutions of stochastic partial integro-differential equations driven by white noise. Numer. Math..

[27] Gunzburger M, Li B, Wang J (2019). Sharp convergence rates of time discretization for stochastic time-fractional PDEs subject to additive space-time white noise. Math. Comp..

[28] Gut, A.: An intermediate course in probability. In: Springer Texts in Statistics, Second edition. Springer-Verlag, New York (2009)

[29] Gyöngy I (1998). Lattice approximations for stochastic quasi-linear parabolic partial differential equations driven by space-time white noise I. Potential Anal..

[30] Hausenblas E, Kovács M (2018). Global solutions to stochastic Volterra equations driven by Lévy noise. Fract. Calc. Appl. Anal..

[31] Jin B, Yan Y, Zhou Z (2019). Numerical approximation of stochastic time-fractional diffusion. ESAIM Math. Model. Numer. Anal..

[32] Karczewska A, Lizama C (2007). Stochastic Volterra equations driven by cylindrical Wiener process. J. Evol. Equ..

[33] Karczewska A, Lizama C (2009). Strong solutions to stochastic Volterra equations. J. Math. Anal. Appl..

[34] Karczewska A, Zabczyk J (2000). Regularity of solutions to stochastic Volterra equations. Atti Accad. Naz. Lincei Cl. Sci. Fis. Mat. Natur. Rend. Lincei (9) Mat. Appl..

[35] Kim I, Kim K, Lim S (2019). A Sobolev space theory for stochastic partial differential equations with time-fractional derivatives. Ann. Probab..

[36] Kovács M, Larsson S, Lindgren F (2012). Weak convergence of finite element approximations of linear stochastic evolution equations with additive noise. BIT.

[37] Kovács M, Larsson S, Mesforush A (2014). Erratum: Finite element approximation of the Cahn-Hilliard-Cook equation. SIAM J. Numer. Anal..

[38] Kovács M, Larsson S, Saedpanah F (2020). Mittag-Leffler Euler integrator for a stochastic fractional order equation with additive noise. SIAM J. Num. Anal..

[39] Kovács M, Lindner F, Schilling RL (2015). Weak convergence of finite element approximations of linear stochastic evolution equations with additive Lévy noise. SIAM/ASA J. Uncertain. Quantif..

[40] Kovács M, Printems J (2014). Strong order of convergence of a fully discrete approximation of a linear stochastic Volterra type evolution equation. Math. Comp..

[41] Kovács M, Printems J (2014). Weak convergence of a fully discrete approximation of a linear stochastic evolution equation with a positive-type memory term. J. Math. Anal. Appl..

[42] Larson, M. G., Bengzon, F.: The finite element method: theory, implementation, and practice. In: Texts in Computational Sciences and Engineering. Springer-Verlag Berlin, Heidelberg (2010)

[43] Lototsky SV, Rozovsky BL (2020). Classical and generalized solutions of fractional stochastic differential equations. Stoch. Partial Differ. Equ. Anal. Comput..

[44] Lubich C (1988). Convolution quadrature and discretized operational calculus. I. Numer. Math..

[45] Lubich C (1988). Convolution quadrature and discretized operational calculus. II. Numer. Math..

[46] Lubich C, Sloan IH, Thomée V (1996). Nonsmooth data error estimates for approximations of an evolution equation with a positive-type memory term. Math. Comp..

[47] McLean W, Thomée V (1993). Numerical solution of an evolution equation with a positive-type memory term. J. Austral. Math. Soc. Ser. B.

[48] Mijena JB, Nane E (2015). Space-time fractional stochastic partial differential equations. Stochastic Process. Appl..

[49] Mittag-Leffler G (1903). Sur la Nouvelle Fonction $$E_{\alpha }(x)$$. Comptes Rendus de l’Academie des Sciences Paris.

[50] Podlubny, I.: MATLAB MLF code, File 8738, In: MATLAB Central File Exchange, Retrieved July 26 (2021)

[51] Printems J (2001). On the discretization in time of parabolic stochastic partial differential equations. M2AN Math. Model. Numer. Anal..

[52] Prüss, J.: Evolutionary integral equations and applications. In: Monographs in Mathematics, vol. 87. Birkhäuser Verlag, Basel (1993)

[53] Sperlich S (2009). On parabolic Volterra equations disturbed by fractional Brownian motions. Stoch. Anal. Appl..

[54] Sperlich S, Wilke M (2010). Fractional white noise perturbations of parabolic Volterra equations. J. Appl. Anal..

[55] Thomée, V.: Galerkin finite element methods for parabolic problems. In: Springer Series in Computational Mathematics, vol. 25 (2nd edition). Springer-Verlag, Berlin (2006)

[56] Wu X, Yan Y, Yan Y (2020). An analysis of the L1 scheme for stochastic subdiffusion problem driven by integrated space-time white noise. Appl. Numer. Math..

[57] Zygmund, A.: Trigonometric series, vol. I, II (2nd edition). Cambridge University Press, New York (1959)

